# Enteropathogenic *E*. *coli* relies on collaboration between the formin mDia1 and the Arp2/3 complex for actin pedestal biogenesis and maintenance

**DOI:** 10.1371/journal.ppat.1007485

**Published:** 2018-12-14

**Authors:** Katrina B. Velle, Kenneth G. Campellone

**Affiliations:** Department of Molecular and Cell Biology, Institute for Systems Genomics, University of Connecticut, Storrs, Connecticut, United States of America; INSERM, FRANCE

## Abstract

Enteropathogenic and enterohemorrhagic *E*. *coli* (EPEC and EHEC) are closely related extracellular pathogens that reorganize host cell actin into “pedestals” beneath the tightly adherent bacteria. This pedestal-forming activity is both a critical step in pathogenesis, and it makes EPEC and EHEC useful models for studying the actin rearrangements that underlie membrane protrusions. To generate pedestals, EPEC relies on the tyrosine phosphorylated bacterial effector protein Tir to bind host adaptor proteins that recruit N-WASP, a nucleation-promoting factor that activates the Arp2/3 complex to drive actin polymerization. In contrast, EHEC depends on the effector EspF_U_ to multimerize N-WASP and promote Arp2/3 activation. Although these core pathways of pedestal assembly are well-characterized, the contributions of additional actin nucleation factors are unknown. We investigated potential cooperation between the Arp2/3 complex and other classes of nucleators using chemical inhibitors, siRNAs, and knockout cell lines. We found that inhibition of formins impairs actin pedestal assembly, motility, and cellular colonization for bacteria using the EPEC, but not the EHEC, pathway of actin polymerization. We also identified mDia1 as the formin contributing to EPEC pedestal assembly, as its expression level positively correlates with the efficiency of pedestal formation, and it localizes to the base of pedestals both during their initiation and once they have reached steady state. Collectively, our data suggest that mDia1 enhances EPEC pedestal biogenesis and maintenance by generating seed filaments to be used by the N-WASP-Arp2/3-dependent actin nucleation machinery and by sustaining Src-mediated phosphorylation of Tir.

## Author summary

Microbial pathogens that rearrange the host actin cytoskeleton have made valuable contributions to our understanding of cell signaling and movement. The assembly and organization of the actin cytoskeleton is driven by proteins called nucleators, which can be manipulated by bacteria including enteropathogenic *Escherichia coli* (EPEC), a frequent cause of pediatric diarrhea in developing countries. After ingestion, EPEC adhere tightly to cells of the intestine and hijack the underlying cytoskeleton to create protrusions called actin pedestals. While mechanisms of pedestal assembly involving a nucleator called the Arp2/3 complex have been defined for EPEC, the contribution of additional host nucleators has not been determined. We assessed the roles of several actin nucleators in EPEC pedestals and found that in addition to Arp2/3 complex-mediated nucleation, the formin mDia1 is a key contributor to actin assembly. These findings highlight the importance of nucleator collaboration in pathogenesis, and also advance our understanding of the molecular and cellular basis of EPEC infection, which is ultimately important for the discovery of new drug targets.

## Introduction

Bacteria and viruses have historically been useful tools for studying the regulation of cytoskeletal dynamics [1], as several intracellular pathogens rearrange host actin into comet tails, which propel them through the cytosol [2] and/or promote their transmission from cell-to-cell [3]. Pathogen motility is frequently driven by activation of the Arp2/3 complex, a ubiquitous actin nucleator, through either bacterial [4, 5] or host [6] actin nucleation-promoting factors (NPFs), although how different classes of nucleators cooperate in cells is not well understood. Enteropathogenic *Escherichia coli* (EPEC) and enterohemorrhagic *E*. *coli* (EHEC) are also capable of reorganizing host actin via the Arp2/3 complex, but these pathogens remain extracellular to form actin-rich protrusions of the plasma membrane called pedestals [7, 8]. Actin pedestals promote “surfing” motility [9, 10], which is important for cell-to-cell spread [11]. Because EPEC and EHEC activate the host actin nucleation machinery from an extracellular location, they represent ideal models for studying the transmembrane signaling mechanisms, cytoskeletal dynamics, and nucleator cooperation that underlie cellular protrusions [12].

To trigger actin pedestal assembly, EPEC and EHEC both translocate effector proteins into the host cell using a type 3 secretion system (T3SS) [13]. One effector, Tir (translocated intimin receptor), adopts a hairpin conformation in the plasma membrane and binds to intimin on the surface of the bacterium, enabling tight attachment of EPEC and EHEC to the plasma membrane [14, 15]. For EPEC, intimin-induced clustering of Tir triggers phosphorylation of tyrosine residue 474 within its cytoplasmic region by host cell kinases from the Abl/Arg, Src, and Tec families [16–21]. Phosphotyrosine 474 binds the adaptor proteins Nck1 and Nck2 [22, 23], which in turn recruit the NPF N-WASP, resulting in actin assembly via the Arp2/3 complex [24, 25]. EHEC-mediated pedestal biogenesis differs from that of EPEC, because it does not rely on tyrosine phosphorylation or Nck1/Nck2 [14, 22]. Instead, EHEC Tir binds host BAR proteins including IRTKS [26] and IRSp53 [27] to recruit an additional bacterial effector protein called EspF_U_ [28, 29], which multimerizes N-WASP to achieve Arp2/3 complex-driven actin assembly [30–32].

EPEC and EHEC pedestals serve several potential pathogenic purposes, ranging from phagocytosis resistance to epithelial colonization [33–36]. Recently, actin pedestals were also shown to allow the formation of large, two-dimensional bacterial aggregates called macrocolonies [11]. A macrocolony encompasses multiple epithelial cells and appears to originate from a single adherent bacterium multiplying and using Arp2/3-mediated actin-based motility to reach and infect neighboring cells. This series of events allows the bacteria to effectively spread infection without dissociating from the epithelium [11].

Although the Arp2/3 complex is a major actin nucleator in cells, it is becoming clear that other types of actin assembly factors, including formins and tandem actin monomer-binding proteins of nucleation, can cooperate [37, 38]. Further, it is becoming increasingly apparent that the formation of actin-based cellular structures and the ability to undergo actin-driven processes, such as motility, depend on multiple nucleators [39–41]. For instance, while lamellipodial protrusions are known to rely on Rac-mediated stimulation of WAVE-family NPFs and subsequent activation of the Arp2/3 complex [42], recent work indicates that important contributions come from the formins mDia1 [43] and FMNL2/3 [44] as well. Despite these findings, the precise mechanisms governing nucleator coordination remain poorly understood.

While the lamellipodium represents a valuable model for studying nucleator cooperation, pathogens have also been found to employ multiple nucleators [45], and therefore have the potential to shed light on how such actin assembly factors collaborate. For instance, it is well established that *Listeria monocytogenes* activates the Arp2/3 complex using the bacterial NPF ActA [5, 46, 47], that *Shigella flexneri* uses the bacterial N-WASP activator IcsA [6, 48], and that vaccinia virus relies on the viral membrane protein A36 to bind the Nck1/2 and Grb2 adaptors [49, 50]. However, recent studies have uncovered additional roles for formin nucleators in actin tails and pathogen-associated membrane protrusions. Specifically, protrusion formation and cell-to-cell transmission of *Listeria* and *Shigella* were observed to be negatively impacted by the knockdown or inhibition of Diaphanous formins [51, 52], suggesting that the formin family of nucleators contributes to the force required for bacterial protrusion into neighboring cells. Furthermore, actin comet tails generated by vaccinia virus were found to rely on the formin FHOD1 in addition to N-WASP and Arp2/3 for actin assembly, motility, and cell-to-cell spread [53]. Formin-mediated actin polymerization was also recently shown to be important for the displacement of septins from vaccinia prior to viral egress [54], although the formin responsible has not yet been identified. Lastly, *Rickettsia parkeri* was observed to undergo a switch in motility from Arp2/3 complex dependence early in infection to formin-mediated motility late in infection [55], employing the bacterial NPF RickA [56, 57] followed by the formin-like nucleator Sca2 [58].

Taken together, these studies reveal that exploitation of several actin nucleators or actin assembly pathways may be necessary for efficient pathogen-driven actin assembly and cell-to-cell transmission. However, the potential contribution of multiple nucleators to EPEC or EHEC pedestal biogenesis, motility, or cell-to-cell spreading has not been addressed. Moreover, since EPEC and EHEC utilize either phosphotyrosine signaling or direct N-WASP multimerization, they represent important model systems for studying how distinct Arp2/3 complex-associated actin assembly pathways may be coordinated with the activities of additional nucleators at the plasma membrane. In the current study, we examined the roles of formins and other actin nucleators in both EPEC- and EHEC-induced actin rearrangements. Our results reveal a phosphotyrosine-specific mechanism of pedestal assembly in which mDia1 contributes to both initiating and maintaining Arp2/3-dependent actin polymerization.

## Results

### Chemical inhibition of formins impairs pedestal formation driven by EPEC Tir but not by EHEC EspF_U_

Because EPEC and EHEC initiate actin assembly using different mechanisms, we aimed to evaluate the contributions of the Arp2/3 complex and other nucleators to each of these signaling cascades. However, EPEC and EHEC have distinct repertoires of effectors and different capacities for infecting cultured cell lines [59, 60]. So in order to directly compare their pedestal assembly pathways, we employed two well-characterized strains, EPEC Y474* (referred to hereafter as EPEC) and KC12+EspF_U_ [11]. EPEC differs from the wild type in that it encodes an HA-tagged version of Tir [22]. KC12+EspF_U_ is an EPEC strain that acts as a surrogate for EHEC because it was engineered to express the EHEC version of intimin, HA-tagged EHEC Tir, and myc-tagged EspF_U_ [28]. Thus, the EPEC and KC12+EspF_U_ strains are isogenic except for their pedestal effectors and can be used to examine the differences in actin assembly pathways.

The Arp2/3 complex is thought to be critical for all pathways of actin pedestal assembly by EPEC and EHEC. RNAi-mediated knockdown of the Arp2/3 complex or overexpression of the N-WASP WCA domain, which has a dominant negative effect by sequestering and/or ectopically activating Arp2/3, reduces pedestal formation by both EPEC and EHEC [24, 61]. N-WASP is essential for EPEC pedestal assembly [25, 61], and although some N-WASP-deficient mouse cells do not support EHEC pedestal assembly [62], others can form pedestals when EHEC Tir and EspF_U_ are either delivered by KC12 or directly expressed in the knockout cells [61]. Therefore, we expect inhibition of either the Arp2/3 complex or N-WASP to completely block or diminish pedestal assembly by EPEC as well as KC12+EspF_U_. The roles of other nucleators, like formins, are unknown in the context of EPEC or EHEC infections.

**Fig 1:**
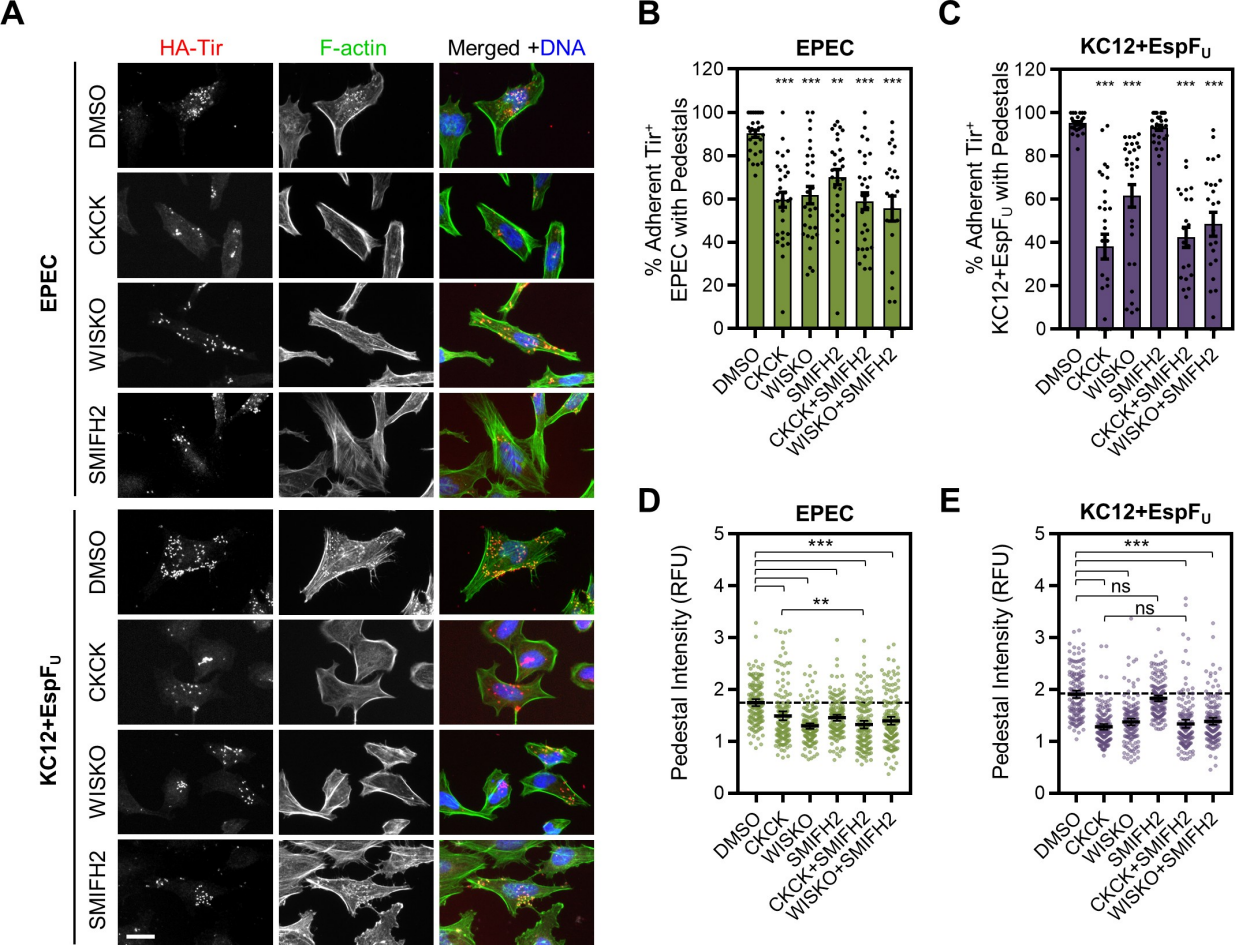
Chemical inhibition of formins decreases actin pedestal assembly by EPEC, but not by KC12+EspF_U_. (A) HeLa cells were pretreated with DMSO, CK666+CK869 (CKCK), Wiskostatin (WISKO), or SMIFH2, and infected with either EPEC or KC12+EspF_U_ for 3.5 h. Cells were fixed and treated with antibodies to detect HA-Tir (red), phalloidin to visualize F-actin (green), and DAPI to stain DNA (blue). Scale bar, 25 µm. (B-C) The % of adherent EPEC or KC12+EspF_U_ (defined by Tir staining) that associated with pedestals was quantified from experiments performed as in A. Each point represents a single infected cell (n = 25–30) harboring 10–50 bacteria, and bars display the mean % +/- SEM. (D-E) The F-actin pixel intensity in the pedestal-forming region (indicated by Tir staining) was quantified and normalized to adjacent pedestal-free areas of the cell, which were set to 1. Each point represents a single EPEC or KC12+EspF_U_ pedestal-forming region, and lines show the mean intensity +/- 95% CI (n = 150 pedestal-forming regions, 15 cells). ** p<0.01, *** p<0.001, ns = not significant (ANOVA, Tukey post-hoc tests). Significance asterisks in B and C are in reference to the DMSO treated conditions.

To initially explore the contributions of the Arp2/3 complex, N-WASP, and formins to actin assembly in pedestals, HeLa cells were pretreated with either DMSO as a control, or the Arp2/3 inhibitors CK666 and CK869 [63], the N-WASP inhibitor Wiskostatin [64], and/or the broad formin inhibitor SMIFH2 [65]. The cells were then infected with EPEC or KC12+EspF_U_ for 3.5 h and stained to detect HA-Tir, F-actin, and DNA (Fig 1A). The fraction of bacteria that translocated Tir and formed a pedestal was assessed, and the F-actin intensity at the locations of HA-Tir staining was quantified and normalized to an adjacent Tir-free area of the cell to determine the relative F-actin levels beneath the bacteria. Treatment with CK666+CK869 caused a 33% reduction in the average percentage of EPEC associated with pedestals, and a 60% reduction in KC12+EspF_U_ associated with pedestals (Fig 1B and 1C). Furthermore, Arp2/3 complex inhibition resulted in significantly dimmer pedestals than DMSO-treated controls for both strains (Fig 1D and 1E). Wiskostatin had similar effects on EPEC pedestals but did not cause as severe of a reduction in the fraction of KC12+EspF_U_ associated with pedestals (Fig 1B and 1C). Collectively, the results from these pharmacological studies are consistent with previous functional analyses demonstrating that N-WASP-Arp2/3 complex-driven pathways of actin assembly are important for pedestal biogenesis.

Interestingly, inhibition of formins using SMIFH2 caused a 20% reduction in the frequency of pedestal formation and significantly dimmer pedestals for EPEC but not for KC12+EspF_U_, which was generally unaffected by SMIFH2 treatment (Fig 1B–1E). Furthermore, the reduction in EPEC pedestal intensity with Arp2/3 complex inhibition was exacerbated by simultaneous formin inhibition (Fig 1D). Other treatment combinations did not strengthen any of the deficiencies in pedestal formation or intensity. These results provide the first evidence, to our knowledge, that formins may be involved in EPEC pedestal assembly.

### Actin-based motility by EPEC is restricted by inhibition of formins

**Fig 2:**
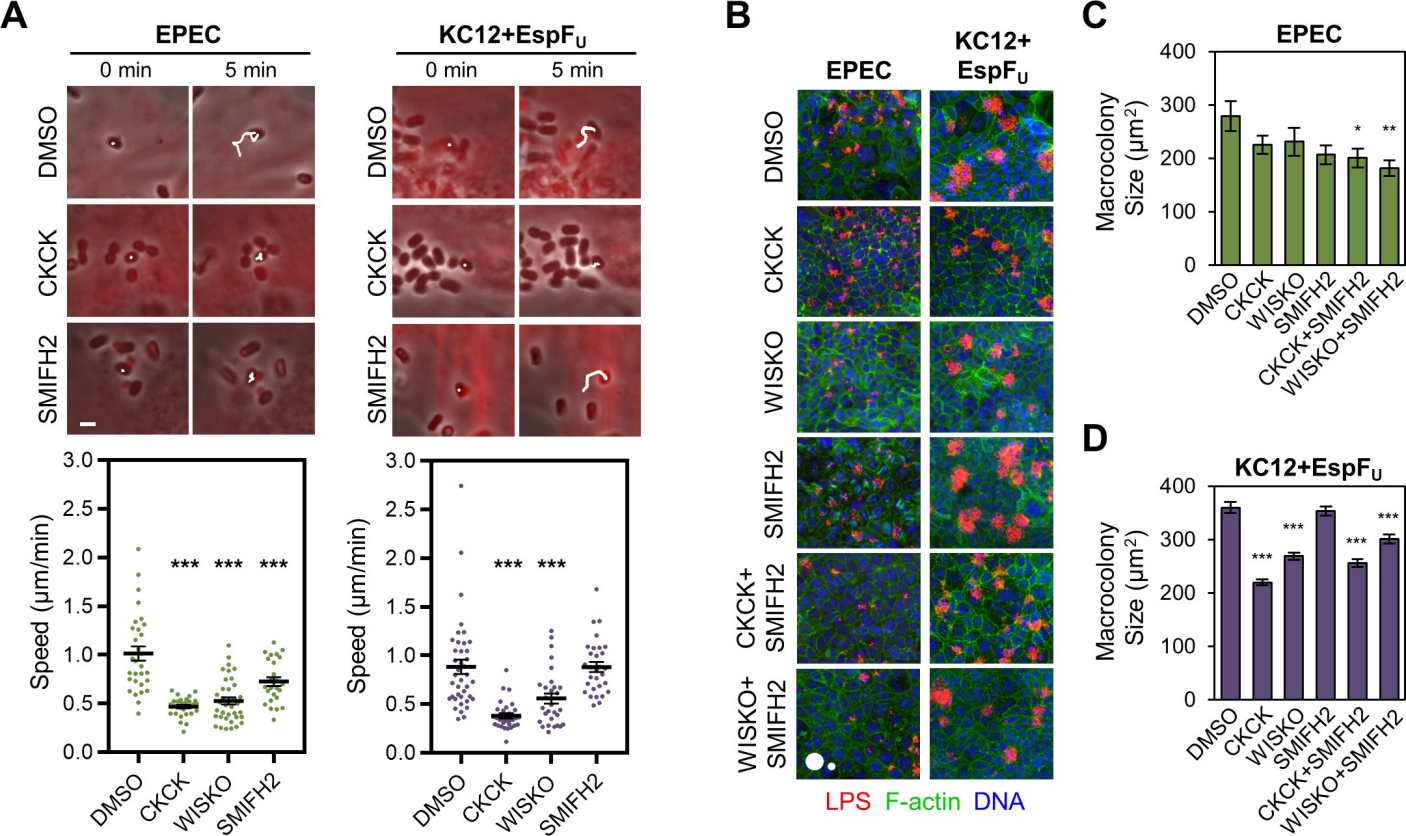
Chemical inhibition of formins impairs EPEC motility and colonization. (A) NIH3T3 cells stably expressing mCherry-actin (red) were infected with EPEC (left) or KC12+EspF_U_ (right) for 3.5–4.0 h, treated with the indicated inhibitors, and imaged live for 20–30 min. Bacteria with pedestals were tracked over time (top panels, scale bar, 2 µm), and white lines highlight the paths taken by representative bacteria during imaging. These experiments were used to determine actin-based motility rates (lower panels). Each point represents a single bacterium associated with a pedestal, and lines show the mean speed +/- SEM (n = 25–40 pedestals, 3–4 cells per condition). *** p < 0.001 (ANOVA, Tukey post-hoc tests). (B) Polarized Caco-2 monolayers were treated with the indicated inhibitors for 15 min prior to and during infection with EPEC or KC12+EspF_U_ for 6 h. Monolayers were then fixed and treated with antibodies to detect LPS (red), phalloidin to visualize F-actin (green), and DAPI to label DNA (blue). Scale circles, 500, 100 µm^2^. (C-D) Experiments shown in B were quantified. Each bar represents the mean macrocolony area +/- SEM (n = 70–124 EPEC colonies, 750–1177 KC12+EspF_U_ colonies). Only macrocolonies larger than 100 µm^2^ were included in the analysis. * p < 0.05, ** p < 0.01, *** p<0.001 (ANOVA, Dunnett’s multiple comparison test). Significance asterisks in A, C, and D are in reference to the DMSO treated conditions.

Actin pedestal-based motility is important for cell-to-cell transmission, and EPEC surfing has been shown to rely heavily on the ability of Tir to become phosphorylated at tyrosine 474 [11], presumably to trigger a Nck1/2-N-WASP-Arp2/3 complex actin polymerization pathway. To determine if SMIFH2 treatment impacts pedestal motility, cells stably expressing mCherry-actin were infected, treated with inhibitors, and subjected to live imaging. Bacteria associated with actin pedestals were tracked over time, and pedestal speeds were calculated using movies spanning 20–30 min. EPEC pedestals moved on DMSO-treated cells at an average speed of 1.02 µm/min, with individual pedestal speeds ranging from 0.40–2.09 µm/min (Fig 2A, left). Treatment with CK666+CK869 reduced the average speed by more than half, to 0.47 µm/min (range: 0.21–0.64 µm/min), and Wiskostatin resulted in a similar reduction in average speed to 0.53 µm/min (range: 0.24–1.10 µm/min). SMIFH2 treatment also significantly inhibited motility, although to a lesser degree, as the average speed was reduced by 28% to 0.73 µm/min (range: 0.34–1.13 µm/min) (Fig 2A, left). Similar to the results in Fig 1, KC12+EspF_U_ motility was only impacted by inhibition of the Arp2/3 complex or N-WASP, and not by SMIFH2 treatment (Fig 2A, right). These results suggest that formin-mediated actin polymerization contributes to the motility of EPEC pedestals, but not EHEC pedestals.

### EPEC colonization is significantly impaired only when both the N-WASP-Arp2/3 complex machinery and formin nucleators are inhibited

Because motility positively correlates with macrocolony size and epithelial colonization [11], we next sought to determine if macrocolony development was impacted by treatment with the various Arp2/3, N-WASP, or formin inhibitors. Polarized Caco2 cell monolayers were thus pretreated with DMSO or inhibitors and infected for 6 h, with hourly washes and media changes to promote colonization only from initially adherent bacteria. Monolayers were then fixed, stained, and imaged at a low magnification to visualize macrocolonies. In line with previous results [11], EPEC consistently formed smaller macrocolonies than KC12+EspF_U_ (Fig 2B). For EPEC infections, treatment with CK666+CK869, Wiskostatin, or SMIFH2 individually limited macrocolony size to some extent, while pairwise combinations of CK666+CK869 with SMIFH2, or Wiskostatin with SMIFH2 resulted in a statistically significant reduction in macrocolony size (Fig 2C). In contrast, KC12+EspF_U_ colonies were unaffected by SMIFH2 treatment, and combining SMIFH2 with either CK666+CK869 or Wiskostatin did not further the deficiencies in colony size beyond what was observed with Arp2/3 or N-WASP inhibition alone (Fig 2D). These data suggest that KC12+EspF_U_ macrocolony size is largely dictated by the N-WASP-Arp2/3 complex pathway of actin assembly, whereas cooperation between the Arp2/3 complex and formins promotes colonization by EPEC.

### Depletion of mDia1 results in a pedestal deficiency that is specific to EPEC

SMIFH2 is a broad inhibitor of actin nucleation by formin FH2 domains [65], so to determine which specific formins could be contributing to EPEC pedestal assembly, we performed a small scale survey of formin function using pairs of siRNAs to the formins that are expressed in HeLa cells, namely DAAM1, FHOD1, FMNL1, FMNL2, INF2, mDia1, mDia2, and mDia3. In addition to targeting these formins, we used siRNAs to the tandem actin monomer-binding proteins of nucleation Cordon-bleu (Cobl), adenomatous polyposis coli (APC), Spire1, and Spire2. We also examined additional Arp2/3 complex interacting proteins in our screen, including Cortactin (CTTN), which was previously reported to contribute to EPEC and EHEC pedestal formation [66, 67], WISH/SPIN90/DIP1, which activates the Arp2/3 complex to promote polymerization of unbranched filaments [68], and JMY, a WASP-family nucleation-promoting factor that can also nucleate actin directly [69]. Lastly, we included two proteins that might influence tyrosine kinase signaling to formins—the GTPase dynamin II (DynII), and the scaffolding protein IQGAP1. DynII contributes to signaling in EPEC pedestals [70], and was recently shown to promote a formin-mediated mechanism of septin displacement from vaccinia virus [54]. IQGAP1 localizes to EPEC pedestals, and actin pedestal formation in IQGAP1-deficient MEFs is reduced by about 40% [71]. Further, *in vitro* experiments suggest that IQGAP1 is capable of binding both to EPEC Tir and to mDia1 [71, 72].

**Fig 3:**
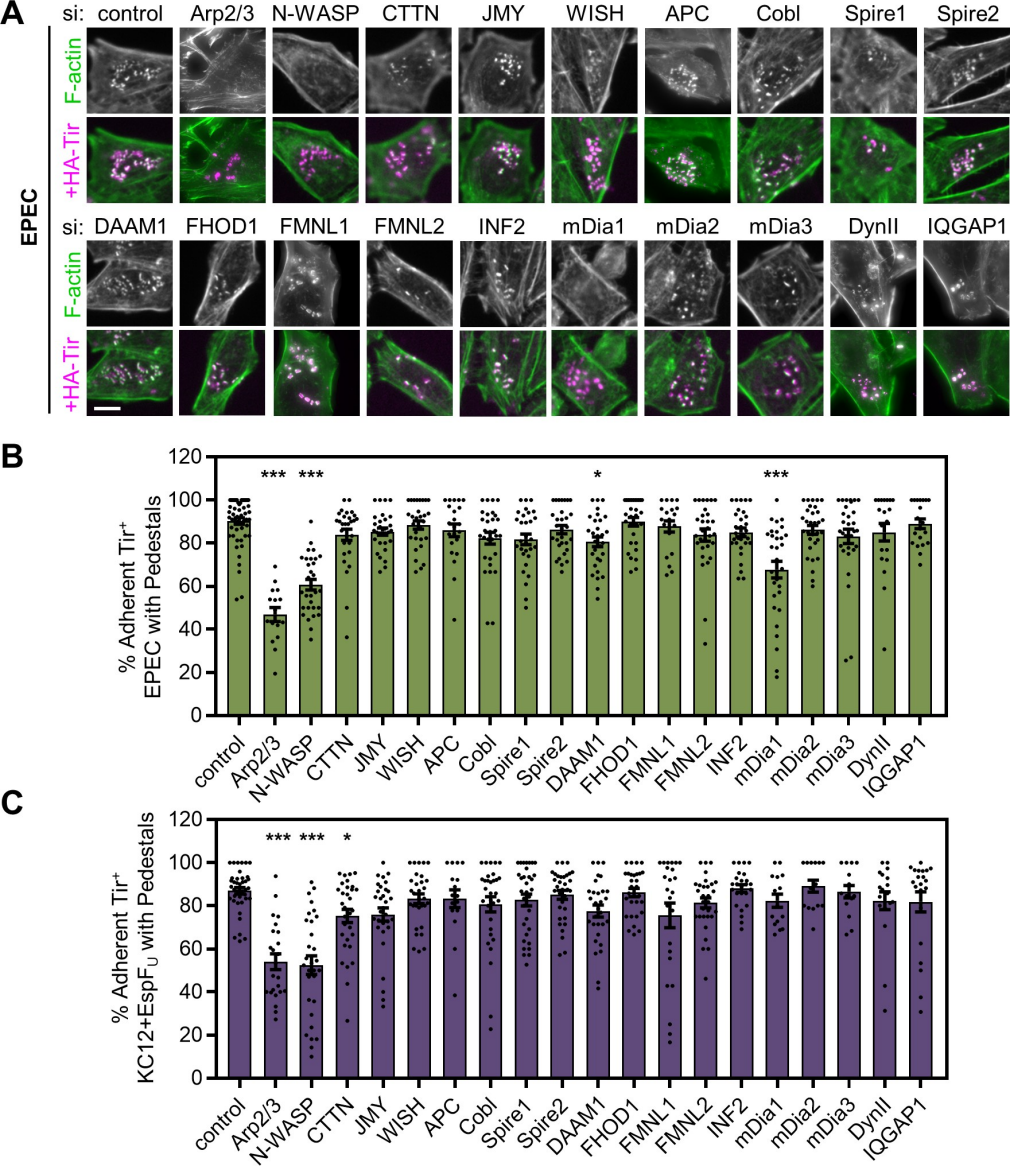
An siRNA screen reveals that targeting mDia1 inhibits actin pedestal formation by EPEC. (A) HeLa cells were treated with siRNA pairs to the indicated targets, infected with EPEC for 4 h, fixed, and stained with antibodies to detect HA-Tir (magenta) and with phalloidin to visualize F-actin (green). Scale bar, 10 µm. (B-C) The % of adherent, Tir-positive EPEC (B) or KC12+EspF_U_ (C) that were associated with pedestals was quantified from experiments performed in A. Each bar shows the mean % +/- SEM of pedestal-forming bacteria, while black data points represent mean %s from each infected cell that harbored 10–50 bacteria (n = 15–53 cells). * p < 0.05, *** p<0.001 (ANOVA, Dunnett’s multiple comparison test). Significance asterisks in B and C are in reference to the control.

On control siRNA-treated HeLa cells, 90% of EPEC and 86% of KC12+EspF_U_ generated pedestals and, as expected, siRNAs targeting the Arp2/3 complex or N-WASP significantly diminished pedestal formation by both strains by 32–48% (Fig 3A–3C). In agreement with previous studies, siRNAs to Cortactin negatively impacted the EspF_U_-dependent pathway of actin polymerization [66], however EPEC pedestals were unaffected. Targeting of JMY, WISH, APC, Cobl, Spire1, or Spire2 did not cause any significant defects in pedestal biogenesis by either strain (Fig 3B and 3C). Among the formins, targeting of DAAM1 resulted in a modest (10%) reduction in EPEC pedestal formation, while targeting of mDia1 (also called DIAPH1 or hDia1) caused a more obvious inhibition of pedestal assembly, reflected in an approximately 25% reduction in pedestal formation frequency (Fig 3B). Although DynII and IQGAP1 would be candidates for promoting an interaction between EPEC Tir and mDia1, we did not observe a measurable defect in pedestal assembly when testing these factors in our screen. Because targeting mDia1 resulted in the same EPEC-specific actin assembly defects that arose with SMIFH2 treatment, we investigated the contributions of mDia1 to EPEC pedestals further.

**Fig 4:**
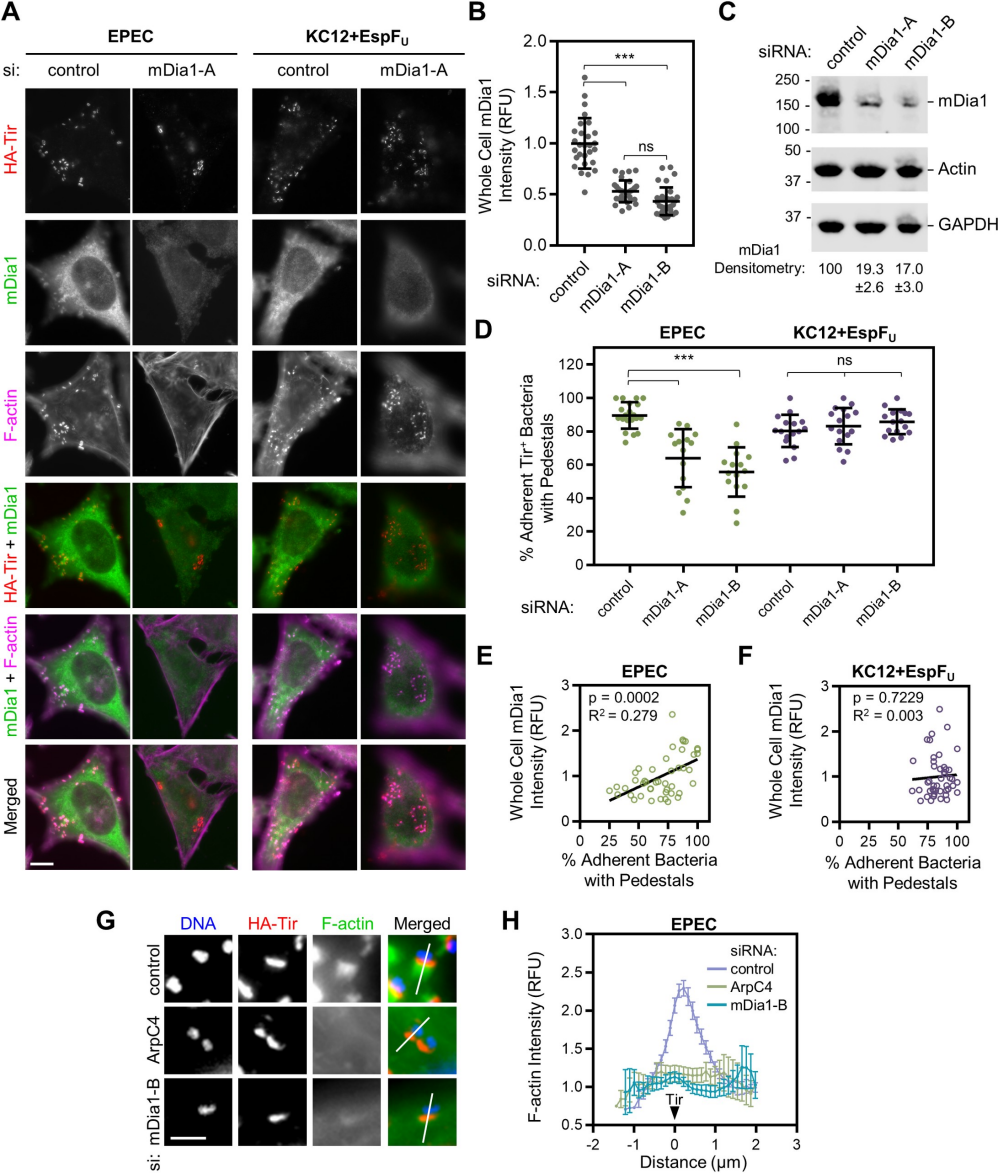
Depletion of mDia1 inhibits actin pedestal assembly by EPEC but not by KC12+EspF_U_. (A) HeLa cells were treated with control siRNAs or independent siRNAs targeting mDia1 and infected with EPEC or KC12+EspF_U_ for 4 h. Cells were fixed and treated with antibodies to detect HA-Tir (red) and mDia1 (green), and with phalloidin to visualize F-actin (magenta). Scale bar, 10 µm. (B) The whole cell fluorescence intensity for mDia1 from experiments shown in A was measured. Each point represents the average pixel intensity of a single cell, and black lines show the mean intensity +/- SD for 28–32 cells. *** p<0.001, ns = not significant (ANOVA, Tukey post-hoc tests). (C) Lysates from uninfected cells treated in parallel to those in A were analyzed by immunoblotting with antibodies to detect mDia1, actin, and GAPDH. mDia1 band intensity was calculated relative to actin and GAPDH and normalized to 100 in control extracts. Data represent the mean +/- SEM quantified from 9 blots encompassing 7 experiments. (D) The % of adherent bacteria (determined by HA-Tir staining) associated with pedestals was quantified from experiments performed in A. Each point represents a single infected cell (n = 15–20 cells) harboring 10–50 bacteria, and lines display the mean % +/- SD. *** p<0.001, ns = not significant (ANOVA, Tukey post-hoc tests). (E-F) The whole-cell intensity of mDia1 staining (for control and mDia1 depleted cells) was plotted against the % of EPEC (E) or KC12+EspF_U_ (F) forming pedestals on that cell. Each point represents a single cell (n = 45–46 cells). Data were subjected to linear regression analysis, and linear trend lines, including R^2^ values, are displayed on the plot with p-values describing whether the slopes are significantly non-zero. (G) Cells were treated with control siRNAs or individual siRNAs targeting ArpC4 or mDia1. After fixation, cells were treated with antibodies to detect HA-Tir (red), with phalloidin to visualize F-actin (green), and with DAPI to label DNA (blue). Scale bar, 2 µm. (H) Pixel intensity plots were generated from cells treated as in G. Lines were drawn through the pedestal-forming region (indicated by Tir staining and displayed as white lines in panel G) and F-actin intensity along the 3 µm line was plotted. All plots were normalized so that a distance of 0 represents the brightest fluorescence of HA-Tir, with the bacteria positioned to the left of 0. Points represent the normalized mean fluorescence of F-actin +/- SEM (n = 21 pedestal-forming regions per condition from 4–5 cells).

Independent siRNAs targeting mDia1 were each effective at depleting cellular mDia1 levels when assessed by immunofluorescence microscopy (Fig 4A and 4B) or western blotting (Fig 4C). Each individual mDia1 siRNA also reduced pedestal formation by EPEC by over 30%, but neither one affected pedestal assembly by KC12+EspF_U_ (Fig 4A and 4D). To more clearly relate cellular mDia1 levels to pedestal formation efficiency, the percentage of EPEC or KC12+EspF_U_ that had successfully formed pedestals on control or mDia1-depleted cells was plotted against the mDia1 staining intensity in those cells (Fig 4E and 4F). For EPEC, the amount of mDia1 present in the cell positively correlated with the percentage of bacteria forming pedestals, but KC12+EspF_U_ formed pedestals more than 60% of the time regardless of mDia1 levels. Finally, to more closely quantify the amount of F-actin that was associated with Tir in mDia1-depleted cells, the phalloidin staining intensity was plotted along a ~3 µm line through the pedestal-forming region, and the brightest pixel in the HA-Tir channel was set to a distance of 0 to compare the intensities across pedestals (Fig 4G and 4H). In control siRNA-treated cells, actin pedestals were strong and peaked immediately adjacent to HA-Tir. However, targeting the Arp2/3 complex or mDia1 diminished this peak in actin intensity, resulting in values that were less than half of those in control cells. These data indicate that EPEC can only assemble pedestals efficiently when mDia1 is present in the host cell.

### mDia1 is preferentially recruited to actin pedestals in a Tir phosphotyrosine-mediated manner

**Fig 5:**
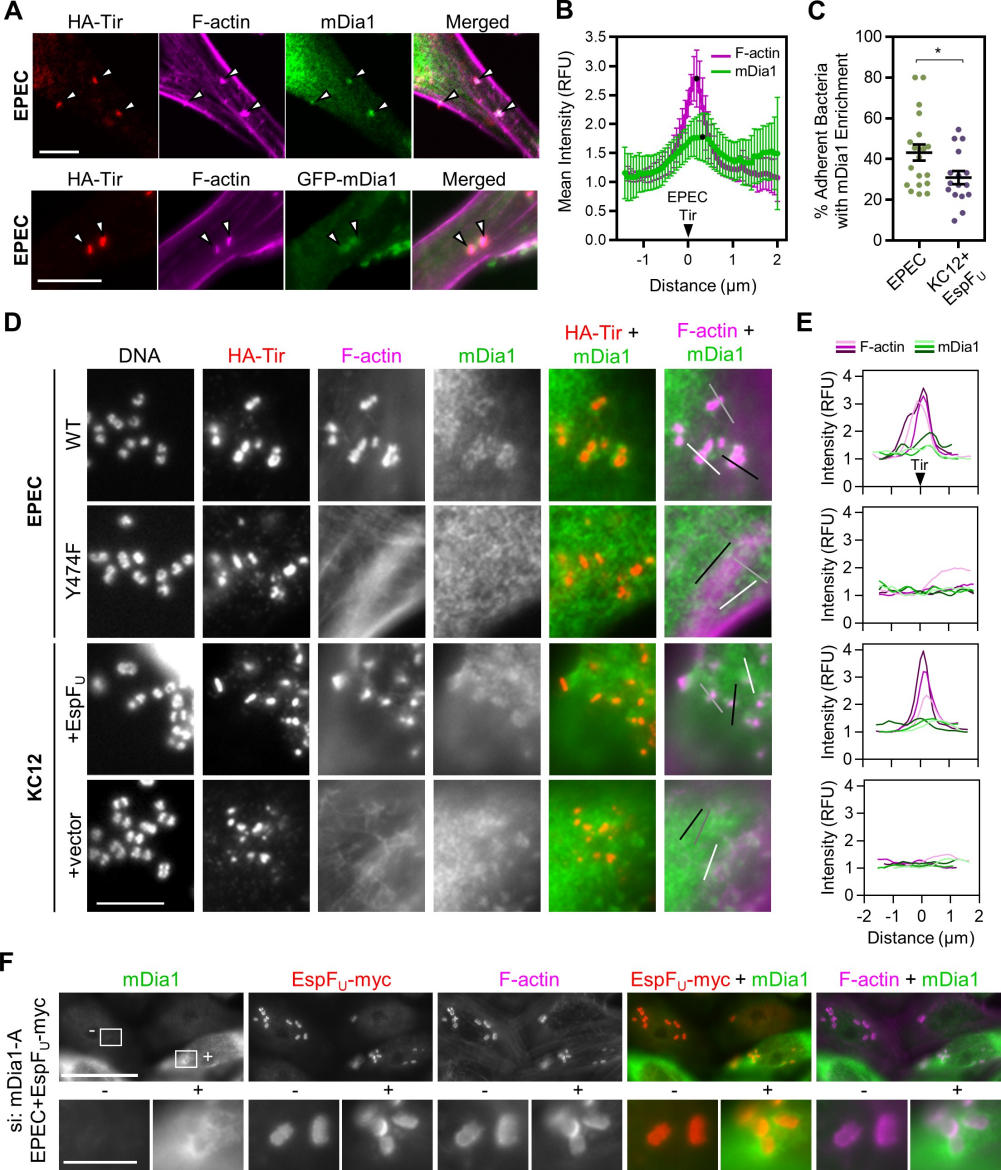
mDia1 is recruited to EPEC pedestals in a Tir phosphotyrosine-mediated manner. (A) HeLa cells (upper panels), or HeLa cells transiently expressing GFP-mDia1 (lower panels) were infected with EPEC for 4 h, fixed, and treated with antibodies to detect HA-Tir, antibodies to stain mDia1 (top panels only), and with phalloidin to visualize F-actin. Arrowheads highlight mDia1-positive pedestals. Scale bars, 10 µm. (B) HeLa cells were infected, fixed, and stained as in A. Lines were drawn through pedestals to measure pixel intensity profiles for HA-Tir, F-actin, and mDia1. The brightest pixel from HA-Tir staining was set to a distance of 0 (indicated by the black triangle) for each pedestal to normalize the F-actin and mDia1 curves (n = 15 pedestals, 4 cells). Each point represents the mean normalized pixel intensity (+/- 95% CI), and black points indicate the maxima. (C) The % of Tir-positive EPEC or KC12+EspF_U_ associated with an enrichment in mDia1 staining was calculated from control siRNA experiments described in Fig 4A. Each point represents a single infected cell, and the mean % +/- SEM is indicated in black (n = 16–20 cells). *p<0.05 (unpaired *t* test). (D) HeLa cells were infected with the indicated strains of EPEC or KC12 for 3.5 h, fixed, and treated with antibodies to detect HA-Tir (red) and mDia1 (green), with phalloidin to visualize F-actin (magenta), and with DAPI to label DNA. Scale bar, 5 µm. (E) 3 adherent bacteria from each panel in D were selected for line scan analysis as in A-B. Each point displays the normalized pixel intensity of F-actin (magenta) or mDia1 (green) along the 2–3 µm line in the corresponding panel. (F) HeLa cells treated with siRNAs against mDia1 were infected with EPEC expressing EspF_U_-myc, then fixed and stained with antibodies to detect mDia1 (green) and EspF_U_-myc (red), and with phalloidin to visualize F-actin (magenta). The “+” indicates a cell with detectable levels of mDia1, and the “-” indicates a cell with substantially less mDia1 because of successful knockdown. Scale bar, 25 µm; inset, 5 µm.

Because mDia1 plays a positive role in EPEC pedestal formation, it would make sense if this protein was present within the pedestal. In fact, endogenous mDia1 could be observed in a subset of pedestals in the control siRNA-treated cells described above (Fig 4A). To more closely assess mDia1 localization, HeLa cells infected with EPEC were fixed and treated with antibodies to detect mDia1 and HA-Tir, as well as phalloidin to visualize F-actin, and examined by confocal microscopy. In parallel, HeLa cells transiently expressing GFP-mDia1 were infected, fixed, and stained for HA-Tir and F-actin. Similar mDia1 recruitment to pedestals was observed with both antibody staining and with the GFP-tagged protein (Fig 5A). To characterize this recruitment more quantitatively, we plotted the pixel intensity profiles of HA-Tir, F-actin, and mDia1 staining along the lengths of several pedestals to determine the position of mDia1 in relation to Tir. On average, F-actin intensity peaked 0.19 µm after Tir, while mDia1 staining peaked 0.13 µm after the F-actin peak (Fig 5B). This implies that the actin associated with mDia1 in pedestals is further from the bacterium than the actin nucleated by Arp2/3 complex, which is thought to localize throughout the pedestal [61] (and see below).

Because mDia1 was not enriched in all EPEC pedestals, we quantified the fraction of pedestals that showed distinguishable mDia1 antibody staining. Additionally, we compared this value to that for KC12+EspF_U_ pedestals, which do not rely on mDia1 during actin polymerization. Pedestals were scored as mDia1 positive or negative, and the average percentage of bacteria in each category was calculated per cell. mDia1 was enriched in 43% of pedestals generated by EPEC and 31% of pedestals formed by KC12+EspF_U_, and this slight preference for EPEC pedestals was statistically significant (Fig 5C).

To determine if mDia1 recruitment was dependent on the known bacterial components that drive pedestal assembly, we employed two pedestal-deficient mutants. For disrupting the main pathway of pedestal biogenesis by EPEC, we used a strain that encodes an HA-tagged version of Tir with a Y474F point mutation that prevents Nck-mediated signaling to the actin assembly machinery (EPEC Y474F) [22]. To interrupt the mechanism used by EHEC, we employed a strain of KC12 lacking EspF_U_ (KC12+vector) [28]. HeLa cells were infected with these bacteria in addition to their pedestal-proficient counterparts, fixed, and stained to visualize HA-Tir, mDia1, F-actin, and DNA (Fig 5D). EPEC expressing wild type (WT) Tir and KC12+EspF_U_ both formed bright pedestals that had some level of mDia1 enrichment, which was reflected in mDia1 pixel intensity profiles associated with adherent bacteria (Fig 5D and 5E). However, EPEC Y474F and KC12+vector did not assemble pedestals or recruit detectable levels of mDia1 (Fig 5D and 5E). These findings suggest that for EPEC, mDia1 recruitment is reliant on a phosphotyrosine 474-dependent pathway of actin polymerization, while for EHEC, EspF_U_-mediated actin assembly can also permit some degree of mDia1 localization to pedestals.

Because KC12+EspF_U_ pedestals can recruit mDia1 but are unaffected by SMIFH2 or mDia1 siRNAs, we reasoned that EspF_U_ could allow the bacteria to bypass a dependency on mDia1. To confirm this possibility, we used a strain co-expressing EPEC Tir and EHEC EspF_U_-myc [11] to infect cells treated with siRNAs targeting mDia1. Cells that avoided knockdown and retained mDia1 expression were easily discernible from nearby mDia1-depleted cells when stained with mDia1 antibodies. Side-by-side comparisons of these mDia1 expressing versus depleted cells revealed that the actin pedestals formed by EPEC+EspF_U_-myc in the presence of mDia1 (Fig 5F, “+” inset; mean fluorescence: 4347 +/-402; n = 15 pedestals, 3 cells), were indistinguishable from the pedestals that these bacteria formed in the absence of mDia1 (Fig 5F, “-” inset; mean fluorescence: 4359 +/-410; n = 15 pedestals, 3 cells). Thus, EspF_U_ is sufficient to confer EHEC’s robust mDia1-independent pedestal-forming ability to EPEC.

### The Arp2/3 complex is essential for all forms of actin pedestal assembly

**Fig 6:**
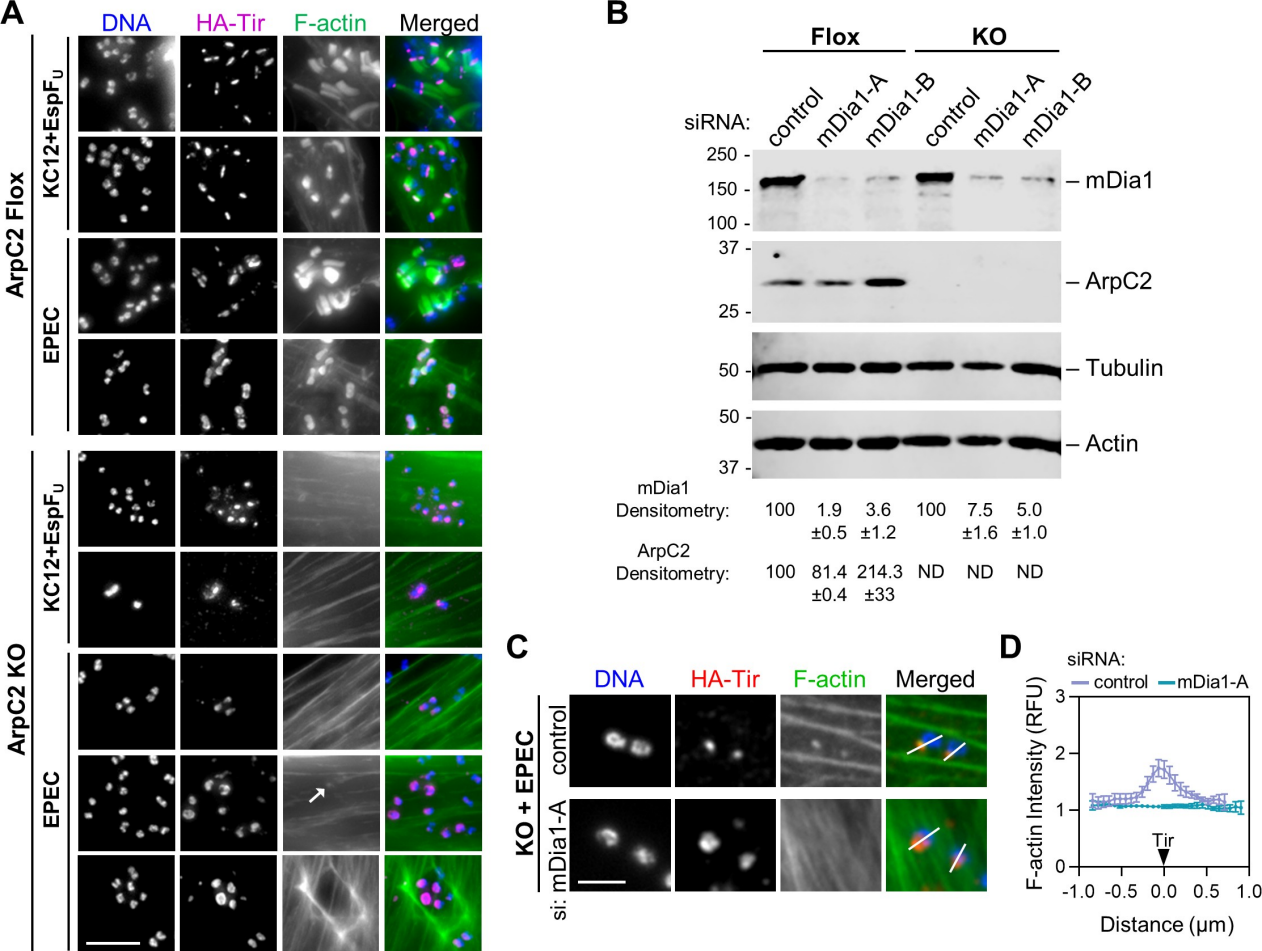
The Arp2/3 complex is essential for all forms of actin pedestal assembly. (A) ArpC2-floxed mouse fibroblasts were treated with DMSO (ArpC2 Flox) or tamoxifen (ArpC2 KO) for 6 days to delete *arpC2* and deplete the Arp2/3 complex. Following 2–6 days of culturing in normal growth media, cells were infected with EPEC or KC12+EspF_U_ for 4 h, fixed, and stained with antibodies to detect HA-Tir (magenta), phalloidin to visualize F-actin (green), and DAPI to label DNA (blue). The arrow denotes a weak actin punctum. The bottom row shows an F-actin basket. Scale bar, 5 µm. (B) ArpC2 Flox and KO cell populations cultured as in A were treated with control siRNAs or independent siRNAs targeting mouse mDia1. Cell lysates were analyzed by immunoblotting with antibodies to detect mDia1, ArpC2, tubulin, and actin. Mean mDia1 and ArpC2 band intensities +/- SEM were normalized to tubulin and actin and quantified from 2 experiments. ND, not detectable. (C) ArpC2 KO cells were treated with control or mDia1 siRNAs, infected with EPEC, fixed, and stained with antibodies to detect HA-Tir (red), phalloidin to visualize F-actin (green), and DAPI to label DNA (blue). Scale bar, 2 µm. (D) Pixel intensity plots were generated from cells treated as in C. Lines were drawn through the pedestal-forming region (indicated by Tir staining and displayed as white lines in panel C) and F-actin intensity along the line was plotted. All lines were normalized so that a distance of 0 represents the brightest fluorescence of HA-Tir, with the bacteria positioned to the left of 0. Points represent the normalized mean fluorescence of F-actin +/- SEM (n = 9 pedestal-forming regions per condition from 3 cells).

We next sought to determine if the contribution of mDia1 to EPEC pedestals is dependent on or independent of the Arp2/3 complex. Because cells treated with chemical inhibitors or siRNAs to target the Arp2/3 complex were still capable of forming pedestals beneath about 50% of bacteria, it was unclear if mDia1 was responsible for this degree of pedestal formation, or if the ability to make pedestals under these conditions was due to residual Arp2/3 complex activity. To differentiate between these possibilities, we infected cells completely lacking the Arp2/3 complex. ArpC2 knockout (KO) mouse fibroblasts [73] were generated by treating ArpC2 Flox cells with 4-hydroxy-tamoxifen for 6 days to delete *arpC2* and fully deplete the Arp2/3 complex. These cells were then compared to DMSO-treated ArpC2 Flox control cells during infection. In striking contrast to the >90% of adherent EPEC and KC12+EspF_U_ which formed pedestals in Arp2/3-proficient cells, 0% of bacteria triggered pedestal assembly in the Arp2/3-deficient cells (Fig 6A, n = 324–344 Tir^+^ bacteria). These results using ArpC2 KO cells indicate that the pedestals which formed during CK666+CK869 or ArpC4 siRNA treatment relied on residual Arp2/3 complex activity, and therefore any effect of mDia1 on EPEC pedestals still requires N-WASP [25, 61] and the Arp2/3 complex.

Although we observed no instances of structures resembling actin pedestals in the KO cells, adherent EPEC were occasionally associated with very small and weak actin puncta (Fig 6A, arrow) or intense F-actin staining in basket-like structures around clusters of translocated Tir (Fig 6A, bottom row). While the origin of the filaments comprising these rare actin baskets remains unclear, we tested whether mDia1 might be responsible for generating the Tir-associated actin puncta by treating ArpC2 Flox and KO cells with mDia1 siRNAs prior to infection with EPEC. Immunoblotting confirmed that the mDia1 siRNAs depleted mDia1 in both cell lines, and that ArpC2 levels were undetectable in the KO cells (Fig 6B). Furthermore, RNAi-mediated depletion of mDia1 prevented the formation of actin puncta in the vicinity of Tir in the KO cells (Fig 6C and 6D). These observations are consistent with the idea that even though mDia1 is insufficient to assemble mature actin pedestals in the absence of the Arp2/3 complex, it is nevertheless capable of promoting some degree of actin polymerization in response to signaling from EPEC Tir.

### mDia1 and the Arp2/3 complex localize to distinct subregions within EPEC pedestals

**Fig 7:**
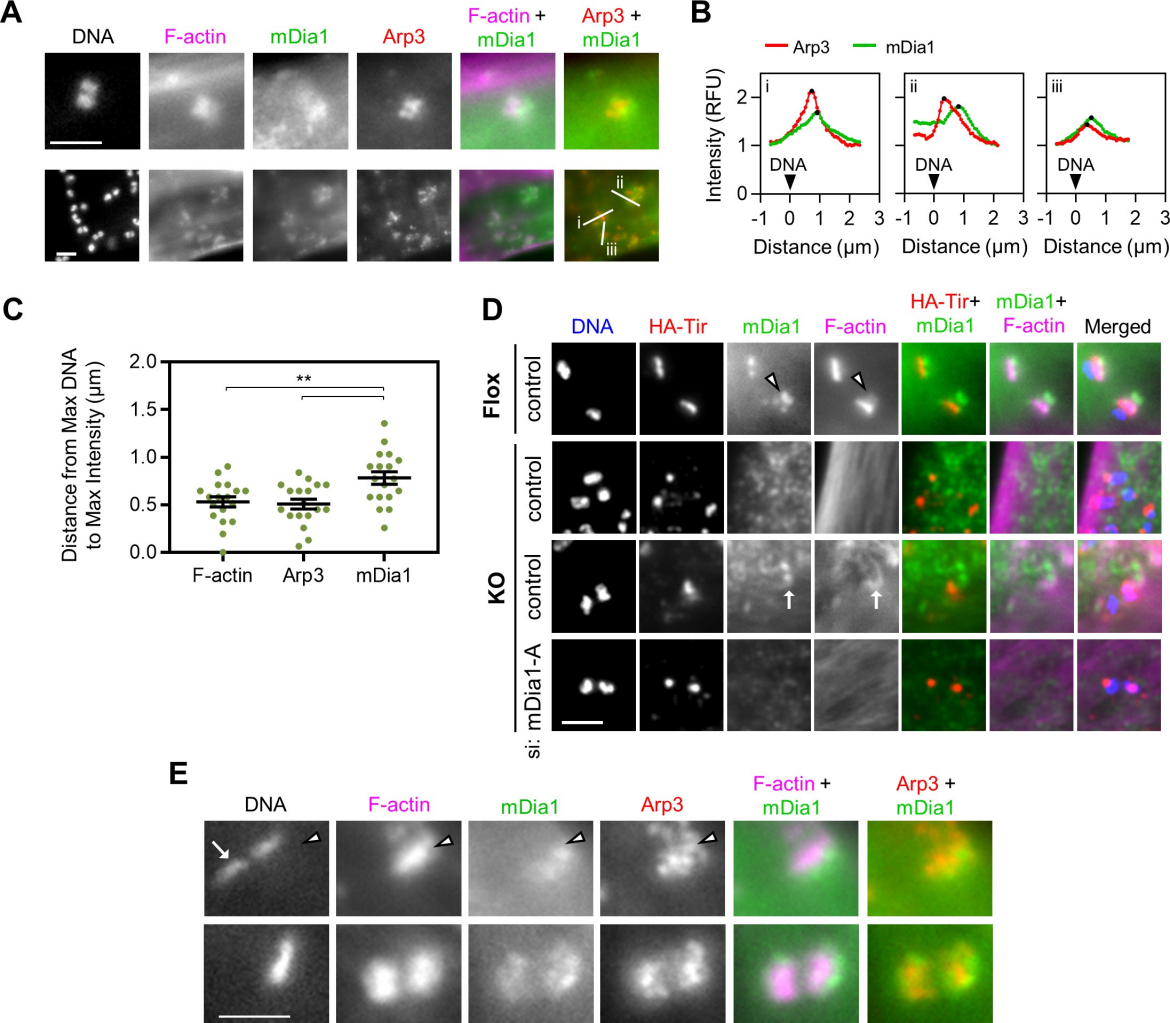
mDia1 and the Arp2/3 complex localize to distinct subregions within EPEC pedestals. (A) HeLa cells were infected with EPEC for 4 h, fixed, and treated with antibodies to detect mDia1 (green) and Arp3 (red), as well as phalloidin to visualize F-actin (magenta), and DAPI to label DNA. Scale bars, 2 µm. (B) Pixel intensity plots were generated from 3 actin pedestals selected from A (white lines i, ii, and iii). Each point represents the relative fluorescence intensity of Arp3 (red) or mDia1 (green) along the 2–3 µm line. All lines were normalized so that a distance of 0 represents the brightest fluorescence of DNA, with the pedestal positioned to the right of 0, and black points represent the maxima. (C) Line scan analyses of EPEC pedestals stained as in A and analyzed as in B were used to calculate the distance from the maximum DNA signal (positioned at 0) to the distance of the maximum signal for F-actin, Arp3, and mDia1 staining. Each point represents the distance within an individual pedestal (n = 18 pedestals), and black lines indicate the mean distance +/- SEM. ** p<0.01 (ANOVA, Tukey post-hoc test). (D) ArpC2 Flox and KO cells were treated with control or mouse mDia1 siRNAs, infected with EPEC, fixed, and stained with antibodies to detect HA-Tir (red) and mDia1 (green), as well as phalloidin to visualize F-actin (magenta), and DAPI to label DNA (blue). The arrow indicates mDia1 enrichment near an accumulation of F-actin adjacent to Tir. Scale bar, 2 µm. (E) HeLa cells were pre-infected for 4 h with EPECΔ*eae*, then washed and challenged with *E*. *coli* expressing intimin for 10 min. Cells were fixed and stained with antibodies to detect mDia1 (green) and Arp3 (red), phalloidin to visualize F-actin (magenta), and DAPI to label bacterial DNA. The arrow points to a bacterium lacking a pedestal, and the arrowhead indicates a pedestal. Scale bar, 2 µm.

Given that the Arp2/3 complex and mDia1 both contribute to EPEC pedestal formation, we next sought to more precisely characterize the localization and timing of their recruitment by EPEC. Therefore, we used immunostaining to visualize Arp3 and mDia1 after HeLa cells were infected with EPEC for 4 h, when most pedestals have reached steady-state in frequency, size, and motility. Consistent with earlier observations (Fig 5B), pixel intensity plots demonstrated that mDia1 was enriched closer to the base of pedestals (Fig 7A and 7B), while Arp3 was found throughout pedestals and was more abundant closer to the bacteria (Fig 7A and 7B). By calculating the distance from the center of the bacteria to the maximum intensities for F-actin, Arp3, and mDia1, we found that, on average, F-actin and Arp3 both peak at 0.5 µm away from the bacteria, while mDia1 peaks at about 0.8 µm from the bacteria (Fig 7C). This pattern of mDia1 recruitment to the less actin-dense distal regions of pedestals was also observed in mouse ArpC2 Flox cells (Fig 7D, arrowhead), demonstrating that the precise localization of this nucleator is conserved in multiple host species and cell types.

To test whether mDia1 is recruited by EPEC Tir in the absence of the Arp2/3 complex, we also stained ArpC2 KO cells for mDia1. In contrast to its strong pedestal base-enriched localization in Flox cells, mDia1 did not localize near Tir in the vast majority of cells (Fig 7D, top KO row). However, in rare instances where weak or unfocused actin was observed near Tir, mDia1 was slightly enriched (Fig 7D, arrow). Such actin recruitment was not found in cells treated with mDia1 siRNAs (Figs 6C and 6D and 7D). These results support a function for mDia1 in the generation of seed filaments that are utilized by the Arp2/3 complex to form *bona fide* pedestals.

Lastly, to examine the temporal arrival of Arp3 and mDia1 during EPEC pedestal assembly, we synchronized the initiation of actin polymerization in infected cells using prime-challenge experiments. HeLa cells were first treated (“primed”) with a mutant EPEC strain that translocates Tir but lacks intimin (EPECΔ*eae*), and then infected (“challenged”) with a laboratory strain of *E*. *coli* expressing intimin (*E*. *coli*+pIntimin). This approach allows for rapid pedestal assembly within minutes of the clustering and tyrosine phosphorylation of Tir that is induced by the intimin-expressing strain [21]. After a 10 minute challenge, cells were fixed and examined microscopically. Both mDia1 and Arp3 localized to pedestals that had formed beneath *E*. *coli*+pIntimin (Fig 7E, arrowhead), but neither protein was found in association with bacteria in the absence of a pedestal (Fig 7E, arrow). As described above, while both proteins were present in pedestals, they did not colocalize, and Arp3 was interspersed in areas with intense F-actin staining that lacked mDia1. These results indicate that mDia1, like the Arp2/3 complex, is recruited to EPEC pedestals very early in their biogenesis, consistent with a role for mDia1 in the initiation of pedestal assembly. When taken together with steady-state localization data, our findings suggest that mDia1 functions in both initiating and maintaining actin assembly in pedestals, but that it does so in a manner that is spatially distinct from the Arp2/3 complex.

### mDia1 is important for Src-family kinase activation and phosphorylation of EPEC Tir Y474

**Fig 8:**
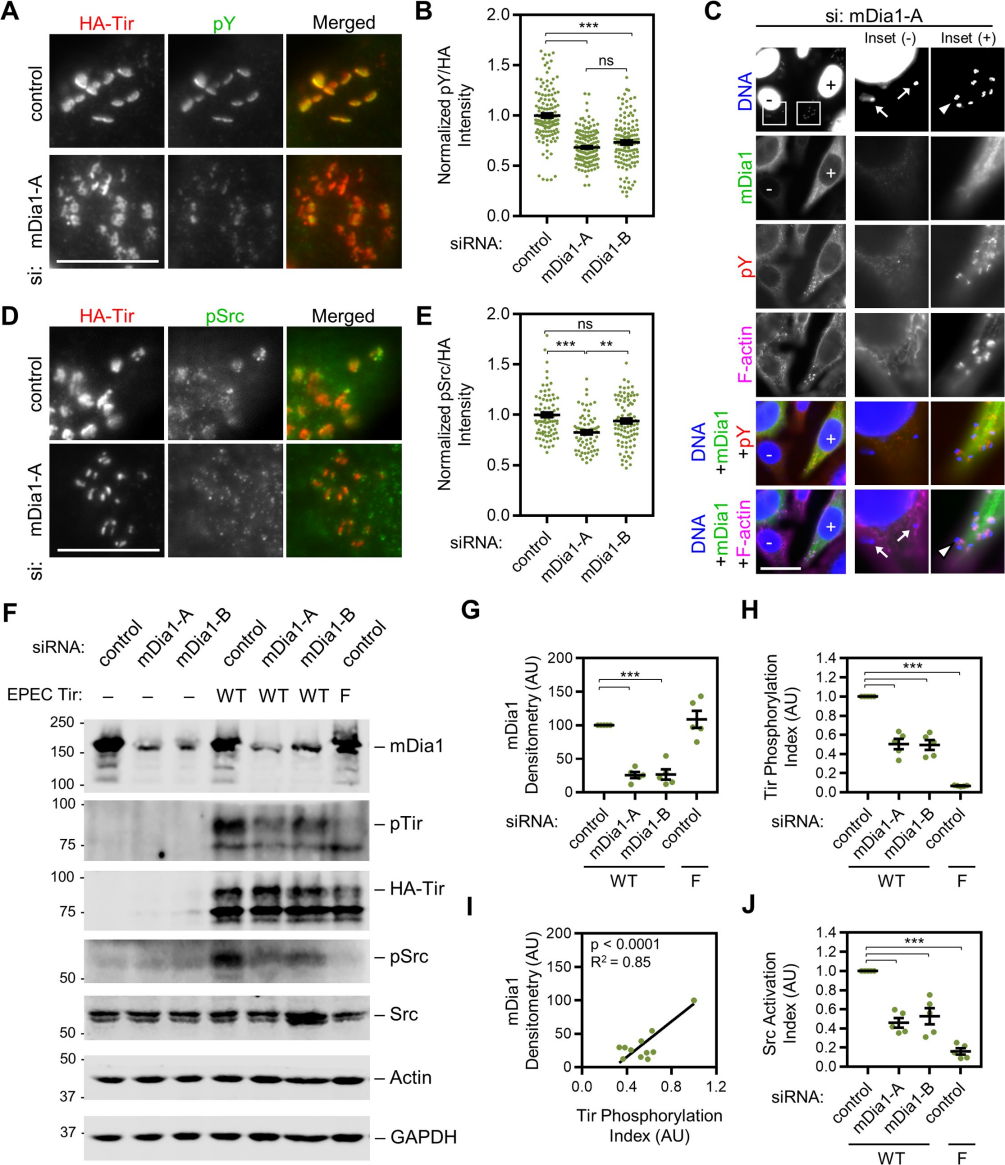
mDia1 is important for Src-family kinase activation and phosphorylation of EPEC Tir Y474. (A) HeLa cells were treated with control or mDia1 siRNAs and infected with EPEC for 4 h. Cells were fixed and stained with antibodies to detect HA-Tir (red) and phosphotyrosine residues (pY, green). Scale bar, 10 µm. (B) The fluorescence intensities of HA-Tir and pY from cells treated as in A were quantified in the pedestal forming regions by outlining the area encompassing HA-Tir and measuring the average pixel intensity within that area. Intensities were normalized to a bacteria-free area of the cell, and then to the mean for control siRNA treated cells, which was set to 1. Each point represents the pY/HA-Tir intensity ratio for an individual bacterium, and black lines show the mean intensity +/- SEM for 131–165 Tir foci from 10 cells per condition. *** p<0.001, ns = not significant (ANOVA, Tukey post-hoc tests). (C) Cells treated with an mDia1 siRNA were fixed and stained with antibodies to detect mDia1 (green) and pY (red), as well as phalloidin to visualize F-actin (magenta), and DAPI to label DNA (blue). The “+” indicates a cell with detectable levels of mDia1, and the “-” indicates a cell with substantially less mDia1 because of successful knockdown. The arrow denotes a bacterium with no pY staining or actin assembly in an mDia1-depleted cell, and the arrowhead highlights a bacterium with pY staining and an actin pedestal in an adjacent mDia1-expressing cell. Scale bar, 25 µm. (D) Cells were treated as in A, but stained with antibodies to phosphotyrosine 416 in active Src-family kinases (pSrc, green). Scale bar, 25 µm. (E) The normalized HA-Tir intensity and pSrc intensity at pedestal forming regions from cells treated as in D were quantified and normalized as in B. Each point represents the normalized pSrc/HA-Tir intensity ratio for an individual bacterium, and black lines show the mean intensity +/- SEM for 70–94 Tir foci from 8 cells per condition. *** p<0.001, ** p<0.01, ns = not significant (ANOVA, Tukey post-hoc tests). (F) HeLa cells were treated with control siRNAs or independent siRNAs targeting mDia1 and either left uninfected (“-”) or infected with EPEC expressing wild type Tir (“WT”) or the Tir Y474F mutant (“F”). Cell lysates were analyzed by immunoblotting with antibodies to mDia1, phosphotyrosine (pTir), HA-Tir, Src phosphotyrosine-416 (pSrc), total Src, actin, and GAPDH. (G) mDia1 band intensities from immunoblots described in F were quantified relative to actin and GAPDH and normalized to the control, which was set to 100. Each point represents the intensity calculated for a single mDia1 band, and the mean +/- SEM is indicated in black. *** p<0.001 (ANOVA, Tukey post-hoc tests). (H) A Tir phosphorylation index was determined from immunoblot band intensities described in F by calculating the pTir to Tir ratio for each condition and normalizing to the control, which was set to 1.0. Each point represents the Tir phosphorylation index within a single sample, and the mean +/- SEM is indicated in black. *** p<0.001 (ANOVA, Tukey post-hoc tests). (I) The mDia1 densitometry points from G were plotted against the Tir phosphorylation indices from H. Data were subjected to linear regression analysis, and the linear trend line and R^2^ value are displayed on the plot, with the p-value indicating that the slope is significantly non-zero. (J) A Src activation index was determined by calculating the pSrc to Src ratio for each condition and normalizing to the control, which was set to 1.0. Each point represents the Src activation index within a single sample, and the mean +/- SEM is indicated in black. *** p<0.001 (ANOVA, Tukey post-hoc tests).

To better understand the mechanisms by which mDia1 contributes to pedestal formation, beyond simply polymerizing actin filaments that could be incorporated into pedestals, we next examined the influence of mDia1 in the signaling pathway that lies upstream of Arp2/3-mediated actin assembly. Because the most fundamental difference between the mechanisms of actin pedestal formation by EPEC and KC12/EHEC is that EPEC relies specifically on phosphotyrosine 474 in Tir, we evaluated the status of Tir phosphorylation in HeLa cells treated with control or mDia1 siRNAs. As expected based on previous quantifications [21, 74], immunofluorescence using antibodies to HA-Tir and phosphotyrosine (“pY”) in control cells revealed that virtually all sites of translocated Tir colocalized with bright phosphotyrosine staining (Fig 8A). In contrast, in cells treated with mDia1 siRNAs, phosphotyrosine colocalization with Tir appeared to be somewhat less frequent, and when it did colocalize with Tir, the intensity of phosphotyrosine staining was noticeably lower (Fig 8A). Quantification of the ratio of pY to HA-Tir intensity in pedestal-forming regions indicated that Tir-associated tyrosine phosphorylation was generally 30% lower in cells treated with independent mDia1 siRNAs compared to cells treated with control siRNAs (Fig 8B). Further, due to cell-to-cell variability in mDia1 silencing, we were able to visualize mDia1-expressing (Fig 8C, “+”) and mDia1-depleted (Fig 8C, “-”) cells in the same field of view. Such images illustrated the fact that bacteria on mDia1-positive cells formed intense actin pedestals containing phospotyrosine and mDia1 (arrowhead), while mDia1-negative cells lacked phosphotyrosine staining and actin pedestal assembly (arrows). These results support an unexpected role for mDia1 in promoting tyrosine phosphorylation of Tir.

Because EPEC-associated tyrosine phosphorylation was influenced by the presence or absence of mDia1, we aimed to test whether mDia1 may also be involved in the activation of the kinases that phosphorylate Tir. Multiple tyrosine kinases can phosphorylate Tir, including Arg, Abl, and Etk [18, 20], as well as the Src-family kinase (SFK) Fyn [17, 19]. Additionally, Src-family members have been shown to interact with several Diaphanous-related formins, including DAAM1, mDia1, and mDia2 [75–78], and mDia1 specifically has been found to affect the subcellular targeting of Src [79, 80]. Therefore, we next probed siRNA-treated cells with an antibody that recognizes active Src-family members, which are characterized by phosphorylation at tyrosine 416 in Src (“pSrc”) or equivalent residues in other SFKs. Consistent with the phosphotyrosine and Tir colocalization results described above, the staining of active SFKs in proximity to Tir was noticeably weaker in cells treated with mDia1 siRNAs than in control cells (Fig 8D). Quantification of the ratio of pSrc to HA-Tir intensity revealed that active SFK levels were indeed lower in the pedestal-forming regions of cells treated with mDia1 siRNAs, although data for one of the siRNAs did not reach statistical significance (Fig 8E).

To further validate our microscopy-based findings on the effects of mDia1 on Tir phosphorylation and SFK activation, siRNA-treated cells that were infected in parallel to those described above were collected, lysed, and subjected to immunoblot analyses for measuring mDia1, pTir, HA-Tir, pSrc, Src, actin, and GAPDH levels (Fig 8F). In accordance with our previous quantifications of mDia1 depletion in uninfected cells (Fig 4C), mDia1 protein levels were reduced by 75%, on average, in infected cells (Fig 8G). Strikingly, even though EPEC delivered the mature 90 kDa form of Tir equivalently into cells treated with different siRNAs, the tyrosine phosphorylation of Tir was clearly lower in the mDia1-depleted samples (Fig 8F). In addition, while infection with EPEC caused a major activation of SFKs in control cells (Fig 8F; compare uninfected lane 1 to infected lane 4; uninfected pSrc:Src ratio = 1, EPEC WT pSrc:Src ratio = 4.99 +/- 0.65, n = 6 blots), this activation was markedly dampened in the mDia1-depleted samples (lanes 5–6). It is important to note that total Src levels were consistent across all conditions and that infections with EPEC expressing the Tir Y474F mutant confirmed both the specificity of Tir tyrosine phosphorylation and its requirement for SFK activation (Fig 8F, lane 7).

Finally, to quantify our biochemical observations, we calculated the relative efficiency of Tir phosphorylation (i.e., Tir tyrosine phosphorylation index) and relative magnitude of Src activation (i.e., Src activation index) across multiple experiments and blots. For assessing Tir phosphorylation, the band intensity of pTir was divided by total HA-Tir. In agreement with our microscopy results, Tir phosphorylation was approximately 50% lower in mDia1-depleted cells than in control cells (Fig 8H). To further evaluate the relationship between cellular levels of mDia1 and phosphorylated Tir, we additionally plotted the mDia1 densitometry data against the Tir phosphorylation index for matched samples, and we found that the two values were positively correlated (Fig 8I). For measuring the relative magnitude of SFK activation in EPEC-infected cells, we calculated the band intensity ratio of pSrc to total Src. We found that SFKs were the most active in infected cells expressing mDia1, significantly less active in infected mDia1-depleted cells, and basically not activated at all when cells were treated with the Y474F mutant instead of wild type Tir (Fig 8J). Collectively, these results indicate that mDia1 is important for SFK activation, which in turn allows for an efficient and persistent phosphorylation of Tir during actin pedestal assembly.

## Discussion

**Fig 9:**
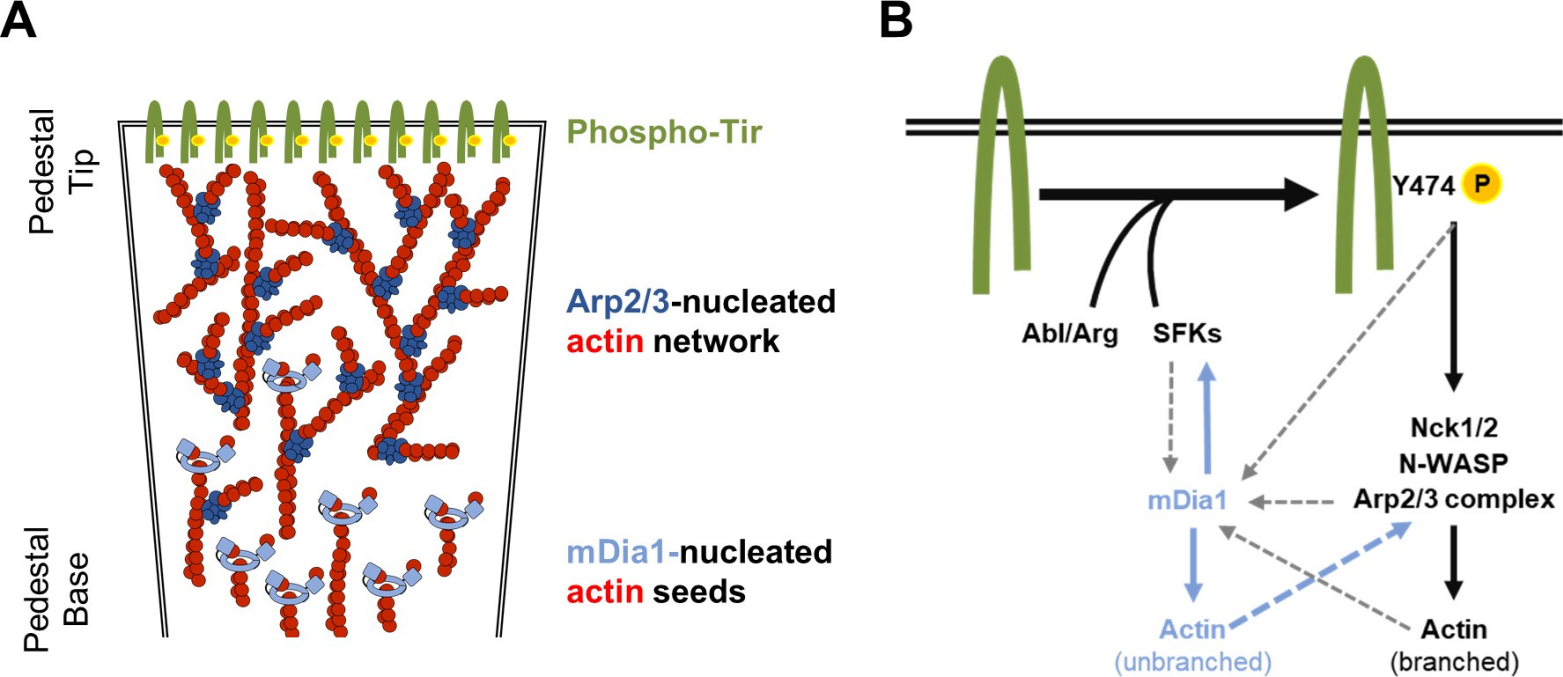
Model for mDia1 functions in EPEC pedestals. (A) We propose that mDia1 activity at the base of the pedestal assembles linear actin filaments, which can be used as mother filaments by the Arp2/3 complex to generate a densely branched network towards the pedestal tip. In the absence of mDia1, pedestals are less numerous and weaker due to fewer seed filaments and lower levels of Tir phosphorylation. In the absence of the Arp2/3 complex, any actin that is nucleated by mDia1 (as weak puncta or actin baskets) is unable to be reorganized and focused into a pedestal. See Discussion for details. (B) A previously established signaling pathway for pedestal formation (black solid arrows) is driven by Tir clustering, tyrosine phosphorylation by Src and Abl family kinases, recruitment of Nck1/2 and N-WASP, and activation of the Arp2/3 complex to polymerize branched actin networks. Our current results expand on this model to include (1) mDia1-associated actin assembly (blue solid arrow) possibly providing seed filaments for the Arp2/3 complex (blue dashed arrow), and (2) mDia1-mediated enhancement of Src activation in pedestals (blue solid arrow) to promote and sustain Tir phosphorylation. It is unclear if mDia1 is recruited by SFKs, pY474, or the Arp2/3 complex, or if Arp2/3-based actin networks that disassemble at the base of the pedestal are recycled by mDia1 (gray dashed arrows). See Discussion for details.

Pathogens such as *Listeria* and *Shigella* are often employed as tools to better understand actin dynamics and uncover new pathways and regulators of actin assembly, yet their utility for modeling actin polymerization at the plasma membrane is limited by the fact that they are cytosolic. By remaining extracellular throughout infection, EPEC and EHEC represent ideal models to study actin rearrangements triggered by transmembrane signaling cascades [12]. While the core pathways of EHEC and EPEC pedestal assembly have been characterized to some degree [81], the potential contributions of actin nucleation factors outside of the Arp2/3 complex and WASP-family have never been directly assessed. Given that the coordinated actions of multiple nucleators orchestrate a variety of cellular functions, including lamellipodia formation and cell motility [39–44], and that both the Arp2/3 complex and formins participate in pathogen-induced protrusions [51–53, 82], we examined whether some form of nucleator cooperation exists in EPEC and EHEC pedestals. Our results indicate that the formin mDia1 contributes to Arp2/3 complex-mediated actin assembly in the pedestals of EPEC but not EHEC. Our findings also support a model in which mDia1 participates in the biogenesis and maintenance of EPEC pedestals by both providing filaments that can be used by the Arp2/3 complex for branched nucleation (Fig 9A) and by promoting tyrosine kinase activation and Tir phosphorylation (Fig 9B).

Our first evidence for formin activity in pedestals came from the use of SMIFH2, which resulted in EPEC-specific pedestal phenotypes, as fewer bacteria formed pedestals, and the pedestals that did form contained less F-actin. These defects are consistent with a role for formins in pedestal biogenesis. Further, SMIFH2 treatment resulted in significantly slower actin-based motility, suggestive of a deficiency in pedestal maintenance and force generation. Lastly, formins also had an apparent role in colonization, as inhibitors of the Arp2/3 complex, N-WASP, or formins did not impact EPEC colony size by themselves, but simultaneously inhibiting both the N-WASP-Arp2/3 machinery and formin nucleators reduced macrocolony size. Taken together, these experiments suggest that collaboration between the Arp2/3 complex and formins is important for pedestal initiation, continuous actin assembly, and EPEC cell-to-cell spreading during colonization.

Using a small siRNA screen, we identified mDia1 as the formin most likely responsible for the EPEC pedestal defects that were observed with SMIFH2. Although targeting DAAM1 also resulted in a decrease in pedestal formation, this phenotype was not as strong or as significant as the one caused by the depletion of mDia1. Further, we found a positive correlation between the cellular level of mDia1 and the fraction of bacteria generating pedestals. These results parallel the findings that *Listeria monocytogenes* and *Shigella flexneri* each rely on Diaphanous formins (mDia1, mDia2, and/or mDia3) in addition to the Arp2/3 complex for protrusion formation and cell-to-cell transmission [51, 52].

Somewhat surprisingly, EPEC did not show any phenotype when FHOD1 was targeted in the siRNA screen. This was unexpected because vaccinia virus, which triggers a similar phosphotyrosine-dependent signaling cascade to EPEC, was found to manipulate Rac1 and FHOD1 for actin tail assembly, motility, and cell-to cell spreading [53]. Canonical vaccinia actin polymerization relies on tyrosine phosphorylation of the viral membrane protein A36 by host cell kinases [49]. Phosphorylated Y112 binds the Nck adaptor proteins, which recruit an N-WASP-WIP complex [50, 83]. Phosphorylation of a second residue, Y132, promotes the recruitment of another adaptor, Grb2, which may contribute to N-WASP activation or stability in the tail [50, 84]. These mechanisms of actin assembly are strikingly similar to the pathways of actin polymerization promoted by EPEC Tir, which is phosphorylated on two similarly spaced residues, Y474 and Y454. Although EPEC does not recruit Grb2 [74], phosphorylated Y474 recruits Nck1 and Nck2, which bind and activate N-WASP, with or without WIP [22, 23, 85]. It is possible that Grb2 somehow promotes FHOD1 recruitment in the case of vaccinia virus, potentially explaining why EPEC does not employ this nucleator. It is also possible that other vaccinia proteins or EPEC effectors positively or negatively influence FHOD1 localization and function. Interestingly, a recent study implicated another formin, which has yet to be identified, in overcoming septin-mediated vaccinia entrapment [54]. Based on our findings, it is plausible that mDia1 is recruited to vaccinia virus and contributes to this ability to evade entrapment and promote egress.

Among pathogenic *E*. *coli*, the formin-related changes in pedestal assembly were exclusive to EPEC, as neither SMIFH2 treatment nor siRNA targeting of formins decreased pedestal formation by KC12+EspF_U_. Despite this, KC12+EspF_U_ was still capable of recruiting mDia1 to pedestals, albeit less frequently than EPEC. Therefore, it seems plausible that mDia1 contributes to EHEC pedestal assembly, but that its effects are dwarfed by the activity of EspF_U_, a multivalent effector protein capable of activating multiple N-WASP molecules to achieve extraordinarily high levels of Arp2/3 complex activation [30–32]. In agreement with this idea, the requirement for mDia1 in EPEC pedestal formation was able to be bypassed by simply introducing EspF_U_ into EPEC.

To understand the mechanisms underlying the contributions of mDia1 to EPEC pedestals, we examined some of the bacterial and host factors that could influence, or be influenced by, mDia1 function. On the bacterial side, we employed the pedestal-deficient strains EPEC Y474F and KC12+vector and found that neither of these mutants efficiently recruited mDia1. This suggests that mDia1 could possibly localize passively to at least a subset of pedestals simply because they are rich in filaments and other actin-associated factors. However, our observation that EPEC Tir-containing pedestals were slightly better than EspF_U_-derived pedestals at recruiting mDia1 leads us to speculate that Tir phosphotyrosine 474 itself, or some signaling molecule associated with this residue, is actively involved in recruiting mDia1. In the course of our siRNA screen, we explored whether several such host cell proteins could mediate mDia1 enrichment in EPEC pedestals. One candidate was WISH, which is capable of interacting with Nck1 and Nck2, as well as mDia1 and the Arp2/3 complex [68, 86, 87]. However, our targeting of WISH with siRNAs did not cause any pedestal phenotypes. Another interesting candidate was IQGAP1, a scaffolding protein that can activate N-WASP and localize to EPEC pedestals [71, 88]. However, our targeting of IQGAP1 in HeLa cells also did not cause any pedestal phenotypes. Whether other factors are able to physically link EPEC Tir to mDia1 in pedestals remains an open question.

In investigating the molecular basis of mDia1 function in EPEC pedestals downstream of Tir Y474, we focused on its ability to polymerize actin and to affect tyrosine kinase signaling. First, to assess its potential cytoskeletal activities, we examined mDia1 localization and actin filaments in the presence and absence of the Arp2/3 complex. In Arp2/3-proficient cells, mDia1 and Arp3 exhibited clearly distinct localization patterns, with Arp3 found throughout pedestals and more abundant closer to the bacteria, whereas mDia1 was concentrated closer to the base of pedestals. In many instances (e.g., Fig 7), F-actin staining was more intense in the Arp3-enriched region, while a weaker F-actin haze was in the more distal mDia1-associated area (most noticeable in Fig 7D, arrowhead). These observations are consistent with the hypothesis that mDia1 may be providing linear seed filaments upon which the Arp2/3 complex can nucleate densely branched actin networks (Fig 9A). In further support of this model are our findings with cells engineered to genetically lack the Arp2/3 complex. In ArpC2 KO cells, EPEC was unable to generate any actin pedestals, confirming the essentiality of the Arp2/3 complex in pedestal assembly. Notably, however, EPEC were sometimes associated with weak F-actin puncta and diffuse mDia1 staining in the KO cells. Such filaments were not observed when mDia1 was depleted, implying that mDia1 can polymerize sparse, unfocused actin filaments near sites of EPEC adherence that could be utilized for branched nucleation if Arp2/3 is present (Fig 9A).

Importantly, the coordination of multiple families of nucleators either directly, as in the case of Spire 1 and Formin 2 *in vitro* [38], or indirectly, as with the multiple assembly factors that operate in lamellipodia [41–44], is an emerging theme in the field of cytoskeletal biology. If mDia1 is indeed providing mother filaments that the Arp2/3 complex can use as seeds for branching and nucleation, the EPEC system might be analogous to studies suggesting that mDia1 and the Arp2/3 complex collaborate by acting sequentially in lamellipodia [43]. Given the fact that we cannot temporally separate the arrival of mDia1 and Arp2/3 at sites of EPEC Tir phosphorylation, our model still requires further investigation. Alternatively, in a fashion similar to mDia2 in the lamellipodia and filopodia of melanoma cells [41], formins may prevent the capping of Arp2/3 complex-nucleated filaments and promote their elongation. However, this seems less likely to take place in the EPEC system due to a lack of mDia1 enrichment at pedestal tips. Lastly, the possibility that both nucleation pathways function independently, in a manner similar to the suggested contributions of FMNL2/3 to Arp2/3-mediated nucleation in lamellipodia [44], is also unlikely due to the complete absence of pedestals in ArpC2 KO cells.

Apart from polymerizing actin filaments that could be incorporated into EPEC pedestals, we also examined whether the molecular mechanism of mDia1 function was related to other activities within pedestals. Tir can be phosphorylated by multiple tyrosine kinases in cells and *in vitro* [17–20], and we focused on the Src family, because Y474 phosphorylation by SFKs is known to be induced by Tir clustering [17, 19]. Diaphanous-related formins have been shown to physically interact with SFKs [75–77], and mDia1 itself has been implicated in the subcellular targeting of Src to the cell periphery or to focal adhesions [79, 80], while another study suggests that Src acts upstream of mDia1 at cell junctions [78]. When we assessed the efficiency of SFK activation and Tir Y474 phosphorylation in control or mDia1-depleted cells, we found that the presence of mDia1 positively correlates with increased SFK activity and Tir phosphorylation, thereby revealing that one of the crucial functions of mDia1 in pedestal formation is to promote SFK activation. Interestingly, EPEC encodes an effector protein, EspJ, that can act as a tyrosine kinase inhibitor [89, 90]. Thus, deciphering the interplay among Tir, mDia1, SFKs, and EspJ during the course of infection will be an important topic for future study.

Building upon several previously-established signaling pathways [81, 91], and in light of our new data, we propose an updated model describing the mechanisms that drive EPEC pedestal assembly (Fig 9B). In the core model: (1) Intimin-mediated clustering of Tir triggers Y474 phosphorylation [16, 21] via Src, Abl, and Tec family kinases [17–20]. (2) This enables Tir binding to the SH2 domains of Nck1 and Nck2 [22, 23]. (3) These adaptors in turn cause N-WASP activation directly via their SH3 domains and linker regions [92–95] or through accessory factors. (4) Active N-WASP then promotes actin branching and nucleation by the Arp2/3 complex to create pedestals [24, 25].

Our current work places mDia1 at several key positions in this model (Fig 9B). First, Y474 induces mDia1 recruitment to the general vicinity of adherent bacteria by a yet-to-be determined mechanism. Since mDia1 does not colocalize with Tir, mDia1 is likely recruited indirectly through a host cell signaling cascade possibly involving SFKs. The actin nucleation activity of mDia1 then assembles unfocused linear filaments that are unable to be organized into a pedestal in the absence of the Arp2/3 complex. However, Nck-mediated activation of the N-WASP-Arp2/3 branching and nucleation machinery results in the repurposing of those seed filaments into *bona fide* actin pedestals, wherein Nck and N-WASP are located in contact with Tir, Arp2/3 is enriched in the proximity of Tir but resides at branches throughout the pedestal network, and mDia1 remains at the distal end of the pedestal. Much like the undefined mechanism of initial mDia1 recruitment, how mDia1 is retained at the pedestal base remains an open question. Nevertheless, the pedestal deficiencies that we observed in mDia1-depleted cells imply that the ability of mDia1 to provide an early and consistent supply of seed filaments is important for building and maintaining pedestals.

Proper pedestal biogenesis and maintenance clearly also rely on the capacity of mDia1 to promote SFK activation and efficient Y474 phosphorylation. Prior to EPEC infection, a housekeeping function of mDia1 may keep SFKs in a proper subcellular environment or activatable form to enable the initial phosphorylation of Tir. Additionally, during the course of infection, it seems likely that mDia1 participates in a positive feedback loop that reinforces SFK activation and sustains Tir phosphorylation in order to provide continuous signaling to Nck, N-WASP, and Arp2/3. The fact that mDia1-depleted cells harbor both fewer pedestals and dimmer pedestals is consistent with mDia1 normally supporting the initial and persistent phosphorylation of Tir and perhaps other pedestal components.

Although our results have provided new insights into actin nucleator collaboration and the cellular mechanisms underlying plasma membrane protrusions by using EPEC as a tool, it is also imperative to revisit the fact that EPEC and EHEC are human pathogens which cause severe diarrheal diseases. Pedestal formation is an important step in EPEC and EHEC pathogenesis, as their abilities to manipulate actin enhances colonization in animal and cell culture models [11, 33, 35, 36]. Therefore, continuing to improve our understanding of the pathways controlling actin assembly could lead to advances in potential therapies. For example, our current findings may renew interest in deciphering how Src-family kinases operate in pedestals and revitalize investigations in the use of tyrosine kinase inhibitors as anti-infectives [96]. In the future, pathogens like EPEC should continue to shed light on how cells normally control actin assembly and how these mechanisms are altered in the context of disease.

## Materials and methods

### Bacterial and mammalian cell culture

All bacterial and mammalian cells are listed in S1 Table. EPECΔ*tir*+pHA-Tir, EPECΔ*tir*+pHA-TirY474F [22], KC12+EspF_U_, KC12+vector [28], EPEC+pEspF_U_-myc [11], EPECΔ*tir*Δ*eae*+pHA-Tir, and *E*. *coli*+pIntimin [21] strains were streaked from glycerol stocks onto LB plates containing 35 µg/ml kanamycin and/or 100 µg/ml ampicillin and used within 2 weeks for host cell infections. 24 h prior to infection, single colonies were grown in LB + antibiotics with shaking at 37°C for 8–9 h. Cultures were then diluted 1:500 in Dulbecco’s Modified Eagle’s Medium (DMEM) + 100 mM HEPES, pH 7.4, with antibiotics and grown standing overnight at 37°C in 5% CO_2_, with the exception of *E*. *coli*+pIntimin, which was grown shaking overnight in LB + ampicillin.

HeLa cells (University of Massachusetts Medical School and University of California, Berkeley, Cell Culture Facility), NIH3T3 cells (University of California, Berkeley, Cell Culture Facility) stably expressing mCherry-βactin, and C2BBe1 (referred to as Caco-2) cells (American Type Culture Collection) were cultured and seeded as described previously [11]. Caco2 cells were maintained with half media changes every 48 h for two weeks post confluency to generate polarized monolayers. ArpC2 Flox mouse fibroblasts (University of North Carolina at Chapel Hill, Bear Lab) [73] were maintained in DMEM (with 4.5 g/L glucose + L-Glutamine + 110 mg/L sodium pyruvate) supplemented with 1x GlutaMAX (Gibco), 10% FBS, and 1x antibiotic/antimycotic (Gibco). To obtain knockout (KO) and control populations, cells were treated with 2 µM 4-hydroxy-tamoxifen (4-OHT) (Sigma) or an equivalent amount of DMSO for 6 days, including a media change to add fresh 4-OHT or DMSO on day 3. ArpC2 Flox and KO cells were returned to normal media and used within a week. All cells were grown at 37°C in 5% CO_2_.

### Infections

Bacterial infections were performed as previously described [11]. Briefly, cells were washed twice with phosphate buffered saline (PBS) and infected with bacteria diluted in DMEM + 3.5% FBS + 20mM HEPES, pH 7.4 to achieve a multiplicity of infection (MOI) of 3–10, depending on the host cell line. For prime-challenge experiments, HeLa cells grown on glass coverslips in 24-well plates were infected with EPECΔ*tir*Δ*eae*+pHA-Tir at an MOI of 6 for 4 h, washed 4 times with PBS, and challenged with ~2 x 10^7^ CFU/ml *E*. *coli*+pIntimin. Immediately following the addition of *E*. *coli*+pIntimin, plates were centrifuged at 172 x g for 5 min, then incubated for 10 min prior to fixation. For generating cell extracts to be used in immunoblotting, HeLa cells grown in 6-well plates were infected with bacteria at an MOI of 20 for 3.5 h, treated with 10 µg/ml gentamicin for 15 min, and washed with PBS prior to collection in PBS + 2mM EDTA and pelleting by centrifugation at 1150 x g for 5 min.

### Chemical inhibitors, RNAi, and transfections

HeLa cells and Caco2 monolayers were treated with 50 µM CK666 + 50 µM CK869 (Calbiochem), 25 µM SMIFH2 (Tocris), 4 µM Wiskostatin (Sigma), or equivalent volumes of DMSO for 15 min prior to infection. During infections, media containing bacteria and inhibitors were added to HeLa cells and Caco2 monolayers, and the latter cells were washed with PBS and given fresh inhibitor-containing media every hour during the course of infection. NIH3T3 cells expressing mCherry-βactin [97] were infected 3.5–4.0 h prior to treatment with inhibitors, and live imaging was completed 15–120 min after the addition of the inhibitors.

RNA and DNA transfections were performed using RNAiMAX or Lippofectamine-LTX reagents (Invitrogen). To clone GFP-mDia1, mDia1 plasmid DNA (variant BC143413, Dharmacon) was PCR amplified as a *Kpn1*-*Not1* fragment using primers ATCATCGGTACCATGGAGCCGCCCGGCGGGAG, and ATCATCGCGGCCGCTTATTAGCTTGCACGGCCAACCAACTC and ligated into the vector pKC-EGFP-C1 [97]. The plasmid was maintained in *E*. *coli* XL-1 Blue. For transient expression, 100 ng of GFP-mDia1 plasmid was transfected in 6-well plates. Sigma MISSION siRNAs (see S2 Table) were used at 40 nM for RNAi experiments. Targets were selected based on HeLa cell expression data cataloged on the Human Protein Atlas (https://www.proteinatlas.org/cell).

### Fluorescence microscopy

Immunofluorescence microscopy was performed as previously described [11], and all antibodies and molecular probes are listed in S3 Table. Briefly, cells seeded onto glass coverslips were fixed in 3.7% PFA for 30 min, washed with PBS, permeabilized with 0.1% TritonX-100, washed, and incubated in blocking buffer (1% FBS + 1% BSA in PBS + 0.02% NaN_3_) for 30 min. Primary antibodies against HA, LPS, mDia1, c-Myc, Arp3, phosphotyrosine, or Src phosphotyrosine-416 were diluted in blocking buffer and cells were probed for 40 min. Cells were washed and treated with Alexa Fluor 488, 555, 568, or 647 conjugated goat anti-rabbit or goat anti-mouse secondary antibodies and/or DAPI and Alexa Fluor 488 or 647 labeled phalloidin for 40 min, followed by washes and mounting in Prolong Gold anti-fade reagent. All fixed and live cells were imaged using a Nikon Eclipse T*i* microscope equipped with Plan Apoλ 100x 1.45 NA, 60x 1.40 NA, and Plan Fluor 20x 0.5 NA objectives, an Andor Clara-E camera, and a computer running NIS Elements software. Live phase-contrast imaging as well as mCherry visualization of infected NIH3T3 cells was performed using the 60x objective, and images were captured at 30 s intervals. A Nikon A1R confocal microscope equipped with a Plan Apo 60X 1.40 NA objective was used to capture the images in Fig 5A. All image processing was completed in ImageJ, and the mTrackJ and Cell Counter plugins were used for analysis. Pixel intensity plots were generated using the “plot profile” tool, and lines were drawn through pedestals or pedestal-forming regions after random selection in the HA-Tir and DAPI channels. Lines were only excluded or shifted if intense F-actin staining from stress fibers interfered with the profile. Statistical analyses of data sets were performed using Graphpad Prism software, and all statistical tests are noted in the figure legends.

### Immunoblotting

To determine the levels of mDia1, ArpC2, pTir, Tir, pSrc, Src, GAPDH, actin, and tubulin, cell pellets collected from 6-well plates were resuspended in lysis buffer (20 mM HEPES pH 7.4, 100 mM NaCl, 1% TritonX-100, 1 mM Na_3_VO_4_, 1 mM NaF, 1mM phenylmethylsulfonyl fluoride, and 10 μg/ml each of aprotinin, leupeptin, pepstatin, and chymostatin), diluted in Laemmli sample buffer, and loaded into 9% SDS-PAGE gels. Proteins were transferred onto nitrocellulose membranes (GE Healthcare), blocked for 30 min in PBS + 5% milk, and exposed to primary antibodies diluted in blocking buffer overnight at 4°C, plus a further 2 h at room temperature. Membranes were rinsed twice with PBS and washed thrice with PBS + 5% Tween-20 (PBS-T). For detecting most primary antibodies, IRDye-conjugated secondary antibodies were diluted in blocking buffer and incubated with the membrane for 1–2 h. For detecting phospho-specific primary antibodies, horseradish peroxidase (HRP-) conjugated secondary antibodies were used. Membranes were again rinsed with PBS and washed with PBS-T. Bands were visualized using a LI-COR Odyssey Fc imaging system. Band intensities were quantified using the Analysis tool in LI-COR Image Studio software. Statistical analyses of data sets were performed using GraphPad Prism software, and all statistical tests are noted in the figure legends.

## Supporting information

S1 Table Bacterial and mammalian cells. (DOCX)

S2 Table siRNAs. (DOCX)

S3 Table Reagents used for immunofluorescence and immunoblotting. (DOCX)
