# Supplementary material for: Enteropathogenic E. coli relies on collaboration between the formin mDia1 and the Arp2/3 complex for actin pedestal biogenesis and maintenance
Source: PLoS Pathog. 2018 Dec 14;14(12):e1007485. doi: 10.1371/journal.ppat.1007485 (PMC6310289; doi:10.1371/journal.ppat.1007485)
Supplement: S1 Table — (DOCX) [file ppat.1007485.s001.docx]

| **Supplementary Table 1.** Bacterial and mammalian cells | |
| --- | --- |
| Bacteria | Reference |
| EPECΔ*tir*+pHA-Tir (“EPEC”) | [22] |
| EPECΔ*tir*+pHA-TirY474F (“EPEC Y474F”) | [22] |
| KC12+pEspF_U_-myc | [28] |
| KC12+vector | [28] |
| EPEC+pEspF_U_-myc (“EPEC+EspF_U_”) | [11] |
| EPECΔ*tir*Δ*eae*+pHA-Tir | [21] |
| *E. coli* (MC1061)+pIntimin | [21] |
| Cells | Reference |
| HeLa (Fig 1, 7, 8A, 8D) | University of Massachusetts Medical School [11] |
| HeLa (Fig 3, 4, 5, 8C) | University of California, Berkeley Cell Culture Facility [97] |
| NIH3T3::mCherry-actin (Fig 2) | [97] |
| Caco-2 BBe1 (Fig 2) | ATCC [11] |
| ArpC2-floxed fibroblasts (Fig 6, 7) | [73] |
