# Supplementary material for: Enteropathogenic E. coli relies on collaboration between the formin mDia1 and the Arp2/3 complex for actin pedestal biogenesis and maintenance
Source: PLoS Pathog. 2018 Dec 14;14(12):e1007485. doi: 10.1371/journal.ppat.1007485 (PMC6310289; doi:10.1371/journal.ppat.1007485)
Supplement: S2 Table — (DOCX) [file ppat.1007485.s002.docx]

| **Supplementary Table 2.** siRNAs | |
| --- | --- |
| Target | Company, Catalog #, Reference, or Sigma siRNA ID |
| None (Universal negative control #1) | Sigma SIC001 |
| ArpC4 | Ambion [97] |
| Arp3 | Ambion [97] |
| N-WASP | A: SASI_HS01_0016243  B: SASI_HS01_0016242 |
| CTTN | A: SASI_HS01_00184662  B: SASI_HS01_00184663 |
| JMY | A: SASI_HS01_00366091  B: SASI_HS01_00081989 |
| WISH | A: SASI_HS01_00075763  B: SASI_HS01_00075762 |
| Cobl | A: SASI_HS01_00131308  B: SASI_HS01_00131307 |
| APC | A: SASI_HS02_00301724  B: SASI_HS02_00329744 |
| Spire1 | A: SASI_HS01_00089951  B: SASI_HS01_00089950 |
| Spire2 | A: SASI_HS01_00138124  B: SASI_HS01_00138125 |
| DAAM1 | A: SASI_HS01_00069723  B: SASI_HS01_00069724 |
| FHOD1 | A: SASI_HS01_00202297  B: SASI_HS01_00202298 |
| INF2 | A: SASI_HS02_00307741  B: SASI_HS02_00307740 |
| FMNL1 | A: SASI_HS01_00192708  B: SASI_HS01_00192710 |
| FMNL2 | A: SASI_HS01_00144237  B: SASI_HS02_00361655 |
| mDia1 | A: SASI_HS02_00313790  B: SASI_HS02_00313791 |
| mDia2 | A: SASI_HS02_00310289  B: SASI_HS02_00310288 |
| mDia3 | A: SASI_HS01_00019994  B: SASI_HS01_00019994 |
| mDia1 * (rat/mouse) | A: SASI_RN02_00228375  B: SASI_RN02_00228382 |
| DynII | A: SASI_HS01_00150975  B: SASI_HS01_00227660 |
| IQGAP1 | A: SASI_HS01_00057473  B: SASI_HS01_00057475 |
| * Since mDia1-B caused a surprising upregulation of ArpC2 in Flox cells (Fig 6B) and NIH3T3 cells (not shown), to avoid any potentially-confounding compensatory changes upon mDia1 targeting, mouse cell phenotypes relied largely on mDia1-A. | |
