# Supplementary material for: Enteropathogenic E. coli relies on collaboration between the formin mDia1 and the Arp2/3 complex for actin pedestal biogenesis and maintenance
Source: PLoS Pathog. 2018 Dec 14;14(12):e1007485. doi: 10.1371/journal.ppat.1007485 (PMC6310289; doi:10.1371/journal.ppat.1007485)
Supplement: S3 Table — (DOCX) [file ppat.1007485.s003.docx]

| **Supplementary Table 3.** Reagents used for Immunofluorescence and Immunoblotting | | | | |
| --- | --- | --- | --- | --- |
| Target | Antibody/Probe | Animal | Concentration | Company & Catalog # |
| *Primary Antibodies (Immunofluorescence):* | | | | |
| LPS (Fig 2) | anti-Lipopolysaccharide | Mouse | 2 µg/ml | Abcam (ab35654) |
| HA-Tir (Fig 1, 3, 4, 5, 6, 7, 8) | anti-HA.11 | Mouse | 1 µg/ml | BioLegend (901501) |
| mDia1 (Fig 4, 5, 7D, 8C) | (polyclonal) anti-DIAPH1 | Rabbit | 1 µg/ml | Abcam (ab11173) |
| mDia1 (Fig 7A, 7E) | (monoclonal) anti-DIAPH1 | Rabbit | 0.66 µg/ml | Abcam (ab129167) |
| Arp3 (Fig 7) | anti-Arp3 | Mouse | 2 µg/ml | Sigma (A5979) |
| EspF_U_-myc (Fig 5) | Anti-c-Myc (9E10) | Mouse | 5.7 µg/ml | Sigma (M4439) |
| pY (Fig 8A) | anti-Phospho-Tyrosine (P-Tyr-1000) MultiMab mix | Rabbit | 1:500 | Cell Signaling Technology (8954) |
| pY (Fig 8C) | anti-Phospho-Tyrosine (P-Tyr-100) | Mouse | 4.2 µg/ml | Cell Signaling Technology (9411) |
| pSrc (Fig 8D) | Phospho-Src Family (Tyr416) (D49G4) | Rabbit | 0.5 µg/ml | Cell Signaling Technology (6943) |
| *Primary Antibodies (Immunoblotting):* | | | | |
| mDia1 (Fig 6, 8) | anti-DIAPH1 (polyclonal) | Rabbit | 1 µg/ml | Abcam (ab11173) |
| mDia1 (Fig 4) | anti-DIAPH1 (monoclonal) | Rabbit | 0.66 µg/ml | Abcam (ab129167) |
| ArpC2 (Fig 6) | anti-ArpC2 | Rabbit | 1 µg/ml | Millipore (07-227) |
| pTir (Fig 8) | anti-Phospho-Tyrosine (P-Tyr-100) | Mouse | 4.2 µg/ml | Cell Signaling Technology (9411) |
| Tir (Fig 8) | HA-Tag (C29F4) | Rabbit | 0.134 µg/ml | Cell Signaling Technology (3724) |
| pSrc (Fig 8) | anti-Phospho-Src Family (Tyr416) (D49G4) | Rabbit | 0.1 µg/ml | Cell Signaling Technology (6943) |
| Src (Fig 8) | anti-Src (36D10) | Rabbit | 0.07 µg/ml | Cell Signaling Technology (2109) |
| GAPDH (Fig 4, 8) | anti-GAPDH | Mouse | 0.04 µg/ml | Proteintech (60004-1-Ig) |
| Tubulin (Fig 6) | anti-Beta-Tubulin | Mouse | 1:10,000 | Developmental Studies Hybridoma Bank (E7) |
| Actin (Fig 4, 6, 8) | anti-Beta-Actin | Mouse | 0.2 µg/ml | Proteintech (66009-1-Ig) |
| *Secondary Antibodies (Immunofluorescence):* | | | | |
| Mouse IgG | Alexa555,568,488,647 anti-mouse | Goat | 4 µg/ml | Life Technologies (e.g. A21424) |
| Rabbit IgG | Alexa555,568,488,647 anti-rabbit | Goat | 4 µg/ml | Life Technologies (e.g. A11034) |
| *Secondary Antibodies (Immunoblotting):* | | | | |
| Mouse IgG | HRP anti-Mouse | Sheep | 1:10,000 | GE Healthcare (NXA931) |
| Rabbit IgG | HRP anti-Rabbit | Donkey | 1:10,000 | GE Healthcare (NA934V) |
| Mouse IgG | IRDye680,800 anti-Mouse | Donkey | 0.05 µg/ml | LI-COR (e.g. 926-32212) |
| Rabbit IgG | IRDye680,800 anti-Rabbit | Donkey | 0.05 µg/ml | LI-COR (e.g. 926-32213) |
| *Other:* | | | | |
| F-actin | Alexa488-Phalloidin |  | 0.2 U/ml | Life Technologies (A12379) |
| F-actin | Alexa647-Phalloidin |  | 0.4 U/ml | Life Technologies (A22287) |
| DNA | 4′,6-diamidino-2-phenylindole (DAPI) |  | 1 µg/ml | Life Technologies (D1306) |
